# Electronic and Structural Properties of MPt_*x*_B_6–2*x*_ (M = Y, Yb): Structural Disorder in an Octahedral Boron Framework

**DOI:** 10.1021/acs.inorgchem.3c01526

**Published:** 2023-11-10

**Authors:** Leonid Salamakha, Oksana Sologub, Berthold Stöger, Gerald Giester, Peter F. Rogl, Herwig Michor, Ernst Bauer

**Affiliations:** † Institute of Solid State Physics, TU Wien, A-1040 Vienna, Austria; ‡ X-Ray Center, TU Wien, A-1060 Vienna, Austria; § Institute of Mineralogy and Crystallography, University of Vienna, A-1090 Vienna, Austria; ∥ Institute of Materials Chemistry, University of Vienna, A-1090 Vienna, Austria; ⊥ Department of Physics of Metals, L’viv National University, 79000 L’viv, Ukraine

## Abstract

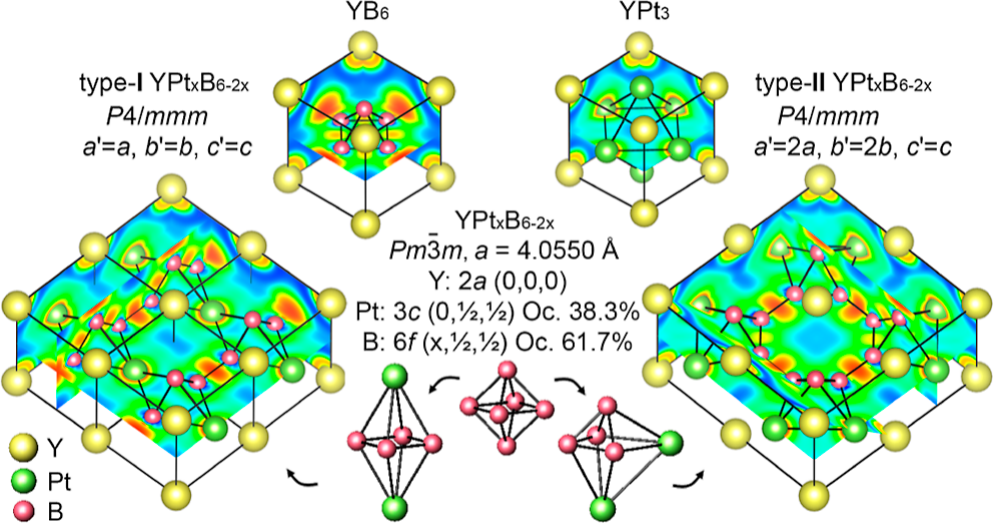

Two new ternary platinum borides, YPt_*x*_B_6–2*x*_ and YbPt_*x*_B_6–2*x*_, were obtained by argon-arc melting of the elements followed by annealing at 780 °C (750 °C). The structures of these compounds combine the fragments of CaB_6_- and AuCu_3_-type structures [space group *Pm*3̅*m*; *x* = 1.15, *a* = 4.0550(4) Å and *x* = 1.34, *a* = 4.0449(2) Å for YPt_*x*_B_6–2*x*_ and YbPt_*x*_B_6–2*x*_, respectively; single-crystal X-ray diffraction]. Two possible variants of B/Pt ordering (space group *P*4/*mmm*) were created via a group-subgroup approach targeting the derived stoichiometry. The architecture of the type-**I** YPt_*x*_B_6–2*x*_ structure model (*a*′ = *a*, *b*′ = *b*, *c*′ = *c*) combines the 4.8^2^ boron nets alternating with the layers of Y and Pt; the type-**II** YPt_*x*_B_6–2*x*_ structure model (*a*′ = 2*a*, *b*′ = 2*b*, *c*′ = *c*) exhibits columns of linked [B_24_] truncated cubes filled with Y running along the *c* axis. The striking features of both structural models are [B_4_Pt_2_] octahedra. The structural similarities with hitherto reported structures (YB_2_C_2_, M_2_Ni_21_B_20_, MNi_21_B_20_, and ErNiB_4_) were drawn supporting the verity of these models. A chemical bonding analysis for type-**I** and type-**II** YPt_*x*_B_6–2*x*_ based on electron localization function distribution revealed a two-center interaction forming the 4.8^2^ boron nets for type-**I** YPt_*x*_B_6–2*x*_ and a covalent bonding within [B_4_Pt_2_] octahedra as well as a two-center interaction for B–B intraoctahedral bonds for type-**II** YPt_*x*_B_6–2*x*_. Analysis of Bader charges revealed the cationic character of the yttrium atoms. The interactions for nondistorted areas of the structures agree well with the bonding picture calculated for constituent building structures, YB_6_ and YPt_3_. Electronic structure calculations predict YPt_*x*_B_6–2*x*_ to be a metal with the density of states of around *N*(*E*_F_) = 1 states eV^–1^ f.u.^–1^. The exploration of the Y–Pt–B system in the relevant concentration range elucidated the homogeneity field of YPt_*x*_B_6–2*x*_ (0.90 ≤ *x* ≤ 1.40) and revealed the existence of three more ternary phases at 780 °C: YPt_2_B (space group *P*6_2_22), YPt_3_B (space group *P*4*mm*), and YPt_5_B_2_ (space group *C*2/*m*).

## Abstract 2

We extended the family of boron-rich compounds to new ternary platinum borides MPt_*x*_B_6−2*x*_ (M = Y, Yb), revealing the structural disorder in the octahedral [B_6_] framework of a hexaboride structure rendered via partial substitution of boron atoms by platinum atoms. Two ordered atomic arrangements (space group *P*4/*mmm*) were considered for structural and bonding analysis of YPt_*x*_B_6−2*x*_. Electronic structure calculations revealed the metallic nature of the compound with a combination of ionic Y–Pt(B), covalent B–B(Pt), and metallic B–Pt interactions.

## 1 Introduction

Boron-based compounds constitute materials with a wide range of mechanical, thermal, chemical, and electrical properties.^1−3^ Among them are hexaborides, showing potential for use in a variety of technical fields (electron emitters, thermoelectric materials, and coatings).^3−12^ Some rare-earth hexaborides also exhibit intriguing magnetic (three distinct magnetic phases in CeB_6_,^13,14^ three-dimensional Kondo topological insulator SmB_6_^15,16^) and superconducting properties (YB_6_ and ThB_6_^17−19^). YB_6_ is a type-**II** BCS superconductor with intermediate or strong electron–phonon coupling, exhibiting relatively high transition temperatures (*T*_c_ = 4.2–8.4 K).^20−23^

The B-rich range of the Y–B system is characterized by the formation of several phases exhibiting three-dimensional boron frameworks,^1^ i.e., icosahedral boron atom frameworks in metal-doped β-rhombohedral B, YB_66_-, YB_50_-, and YB_25_-type compounds; cubo-octahedral framework in dodecaborides of UB_12_-type; and octahedral framework in CaB_6_-type borides. In contrast to icosahedral boron-rich binary structures, for which the generation of structural defects is very complex (e.g., vacancies, partial occupancies of specific atomic sites, and structural distortions occurred due to substitution of boron by foreign atoms),^1,24−34^ the mechanism of formation of CaB_6_-type hexaboride^35^ based homogeneity ranges was found as relatively straightforward, i.e., the deviations from stoichiometry and defect structures for some rare-earth and Th are realized via cation defects or local boron deficits;^4,36,37^ no homogeneity range has been observed for YB_6_.^36^

The unit cell of the hexaboride structure (space group *Pm*3̅*m*, CaB_6_-type) is composed of a single metal atom surrounded by eight [B_6_] octahedra condensed via exohedral B–B bonds. The boron framework is inherently electron deficient and cannot exist without electrons donated from the metal atoms, resembling, in this respect, the clathrate structure.^38^ Because of the small yttrium atomic radius, YB_6_ is located at the border of the structure-type stability.^39^

In general, the hexaborides of the CaB_6_ type are refractory compounds with high melting temperatures (above 2600 °C for YB_6_); they are characterized by excellent acid and oxidation resistance at high temperature and high hardness and high bulk moduli. In recent years, a large number of experimental and theoretical research focused on studying the properties of hexaborides under pressure, revealing the rich chemistry of B in the high pressure polymorphs of hexaborides.^20,40−43^ Several supercells with reduced symmetry for YB_6_ have been claimed to exist at ambient conditions [both from experimental studies (Raman spectroscopy) and DFT calculations] and have been suggested to be more stable than the commonly accepted CaB_6_-type structure.^44−47^

Hexaborides readily form solid solutions with one another;^4^ moreover, the distribution of metal atoms throughout the boron sublattice have been proposed for certain systems, i.e., for 21 K superconductor ThPd_*x*_B_6–2*x*_ (space group *Pm*3̅*m*, *a* = 4.2 Å, *x* = 0.65)^48−50^ and nonsuperconducting YPd_1.2_B_3.3_ [cubic, *a* = 4.2 Å, with an incommensurate modulation vector ***q*** = 0.285 (a* + b* + c*)].^51^ Lately, the solubility of Pd in ThB_6_ has been explored employing the WDX-EPMA and X-ray powder diffraction data of alloys annealed at 950 °C;^52^ a continuous solid solution Th_1–*y*_Pd_*x*_B_6–2*x*_ terminating at composition Th_17.2_Pd_9.0_B_73.8_ (in at. %) was found to form which approximately corresponds to that reported for the 21 K superconductor ThPd_*x*_B_6–2*x*_.^48−50^ Actually, no significant alterations of lattice parameters at about 10 at. % Pd solubility for the solid solution Th_1–*y*_B_6_ (0 ≤ *y* ≤ 0.22)–Th_1–*y*_Pd_*x*_B_6–2*x*_ (*x* ≤ 0.65; 0 ≤ *y* ≤ 0.22 for *x* = 0) (*a* = 4.110–4.115 Å) in comparison with Th_1–*y*_B_6_ (*a* = 4.112 Å) were observed from Rietveld refinement of powder X-ray diffraction data of three phase alloys.^52^ Since the accurate boron content and boron atom positions are difficult to be refined unambiguously from high-resolution electron microscopy, electron diffraction, (WDX-) EPMA, and X-ray powder diffraction data in boride structures that consist of heavy metal atoms next to light boron atoms, further precise studies of MPd_*x*_B_6–2*x*_ or related systems employing single-crystal X-ray diffraction were extremely desired. Furthermore, electronic properties of hexaborides originate from the crystal structure, in which the covalently bonded boron atoms surround the metal atom, which donate electrons to the charge-deficient boron framework; it has been shown by band gap calculations that very low levels of doping cause the changes in B–B intra- and interoctahedra distances and affect the electronic properties.^4^ Thus, considering also the fact that CaB_6_ is, in principle, a structure allowing a relatively high superconducting temperature, understanding the electronic structure and bonding in the family of compounds exhibiting the incorporation of metal atoms into the octahedral boron framework is very important.

Our earlier studies of the ternary Y–Pt–B system^53^ were devoted to structural investigation of the YPt_2_B compound^54^ (CePt_2_B-type structure^55^) and Pt-doped yttrium boride of the YB_50_ family, YB_45–*x*_Pt_*y*_ (space group *Pbam*).^34^ Further careful examination of the phase equilibria in the B-rich corner led to the discovery of a new phase, YPt_*x*_B_6–2*x*_. The isotypic structure has been found in the Yb–Pt–B system. The current paper reports on the crystal structures of these new compounds derived from single-crystal X-ray diffraction. A focus was placed on the bonding and electronic properties, which we elucidated via detailed density functional theory calculations for YPt_*x*_B_6–2*x*_. To understand the obtained structural arrangement, an analysis of the chemical bonding for two ordered structural models created via the group-subgroup approach [hereinafter referred to as type-**I** and type-**II** YPt_*x*_B_6–2*x*_ (structure) models] was performed in comparison with the bonding situation derived for basic structures (YB_6_ and YPt_3_), applying the electron localization function distribution and Bader charges calculations. We emphasize that the type-**I** and type-**II** structure models have been developed to enable the calculation, evaluation, and analysis of the electronic density of states, band structure, and bonding in the disordered YPt_*x*_B_6–2*x*_ structure. With respect to the crystal structure itself, the constructed type-**I** and type-**II** YPt_*x*_B_6–2*x*_ models represent two of the many possible local atomic arrangements within the real crystal structure. Furthermore, the phase relations in the relevant concentration range of the Y–Pt–B system at 780 °C also became the subject of the present work. The results obtained on Y(Yb)–Pt–B systems might be applicable to related ternary boride system, for which the detailed investigation of the phase relations is still pending as well as they will have significant implications for identification the crystal structures and bonding evaluation in ternary phases within MPt_3_-MB_6_ pseudobinary systems, particularly those forming extended antiperovskite type solid solutions and/or a double perovskite-like structure (e.g., in Sc–Pt–B system; author’s unpublished data, the work is in progress).

## 2 Experimental Section

### 2.1 Synthesis and Phase Analysis

Alloys were prepared from pure elements (Pt foil 99.99 mass %, Ögussa, Austria; crystalline boron 99.8 mass %, ChemPur, Germany; Y and Yb pieces 99.9 mass %, ChemPur, Germany) by repeated arc melting under argon. Samples were wrapped in tantalum foil and vacuum-sealed in a quartz tube for annealing at 780 °C (750 °C) for 720 h. The annealed samples were polished by applying standard procedures and were examined by scanning electron microscopy (SEM) using a Philips XL30 ESEM with an EDAX XL-30 EDX-detector to determine Y/Pt ratios. X-ray powder diffraction patterns were collected from annealed alloys employing a Guinier-Huber image plate system with monochromatic Cu Kα_1_ radiation (8° < 2θ < 100°). Quantitative Rietveld refinements of the X-ray powder diffraction data were performed with the program FULLPROF^56^ with the use of its internal tables for atom scattering factors.

### 2.2 Crystal Structure Determination from Single-Crystal X-ray Diffraction Data

For single crystal X-ray diffraction, the crystals were isolated via mechanical fragmentation of the annealed samples. X-ray single crystal intensity data were collected on a four-circle Nonius Kappa diffractometer (CCD detector and graphite monochromated Mo Kα radiation) (YbPt_1.34_B_3.33_) and a Bruker APEX II diffractometer (CCD detector, κ-geometry, and Mo Kα radiation) (for YPt_1.15_B_3.70_). Orientation matrices and unit cell parameters were derived with the help of the diffractometers’ software; the data were scaled using the multiscan approach implemented in SADABS.^57−59^ The structures were solved by direct methods and refined with the SHELXS-97 and SHELXL-97 programs,^60,61^ respectively. Further details concerning the single crystal X-ray diffraction experiments are summarized in Table 1. Detailed descriptions of structural refinements are given below.

**Table 1:** Structure Refinement Details from Single-Crystal XRD of MPt_*x*_B_6–2*x*_ (X = Y, Yb; Space Group *Pm*3̅*m*, No. 221; *Z* = 1; Mo K_α_ Radiation)^a^

compound	YPt_x_B_6–2_*_x_*, *x* = 1.15	YbPt_*x*_B_6–2_*_x_*, *x* = 1.34
nominal composition (at. %)	Y_17.1_Pt_19.6_B_63.3_	Yb_17.6_Pt_23.7_B_58.7_
theta range (deg)	5.03 ≤ θ ≤ 32.70	5.04 ≤ θ ≤ 35.18
crystal size (μm)	54 × 50 × 52	55 × 60 × 58
*a* (Å)	4.0550(4)	4.0449(2)
formula from refinement	YPt_1.15_B_3.70_	YbPt_1.34_B_3.33_
diffractometer	Bruker APEX II	Nonius Kappa
reflections collected/unique	1564/44	108/50
reflections in refinement	44*F*_o_ > 4σ(*F*_o_)	50*F*o > 4σ(*F*_o_)
number of variables	5	5
reliability factors^b^	*R*_F_^2^ = 0.0284	*R*_F_^2^ = 0.0315
	*R*_Int_ = 0.0265	*R*_Int_ = 0.0074
	GOF = 1.388	GOF = 1.353
extinction (Zachariasen)	0.054(1)	0.051(9)
**M1**	1a (0,0,0)	1a (0,0,0)
Occ.	1.00 Y	1.00 Yb
*U*_11_^c^= *U*_22_ = *U*_33_; *U*_23_ = *U*_13_ = *U*_12_ = 0	0.0372(12)	0.0338(8)
**M2**	3c (0,1/2,1/2)	3c (0,1/2,1/2)
Occ.	0.383(3) Pt	0.445(3) Pt
*U*_iso_^d^	0.0071(7)	0.0090(5)
**B1**	6f (*x*,1/2,1/2)	6f (*x*,1/2,1/2)
	*x* = 0.215(5)	*x* = 0.207(6)
Occ.	0.617(3) B	0.555(3) B
*U*_iso_^d^	0.0071(7)	0.0090(5)
residual density (max; min, e–/Å^3^)	0.997; −2.401	1.216; −1.378

a Crystal structure data are standardized using the program Structure Tidy.^62^

b *R*_F_^2^ = Σ|*F*_0_^2^ – *F*_c_^2^|/Σ*F*_0_^2^.

c Anisotropic (*U*_*ij*_) atomic displacement parameters (Å^2^).

d Isotropic (*U*_iso_) atomic displacement parameters of partially occupied atomic sites in YPt_*x*_B_6–2*x*_ and YbPt_*x*_B_6–2*x*_ were constrained to the same values.

#### 2.2.1 YPt_1.15_B_3.70_ and YbPt_1.34_B_3.33_

**Figure 1:**
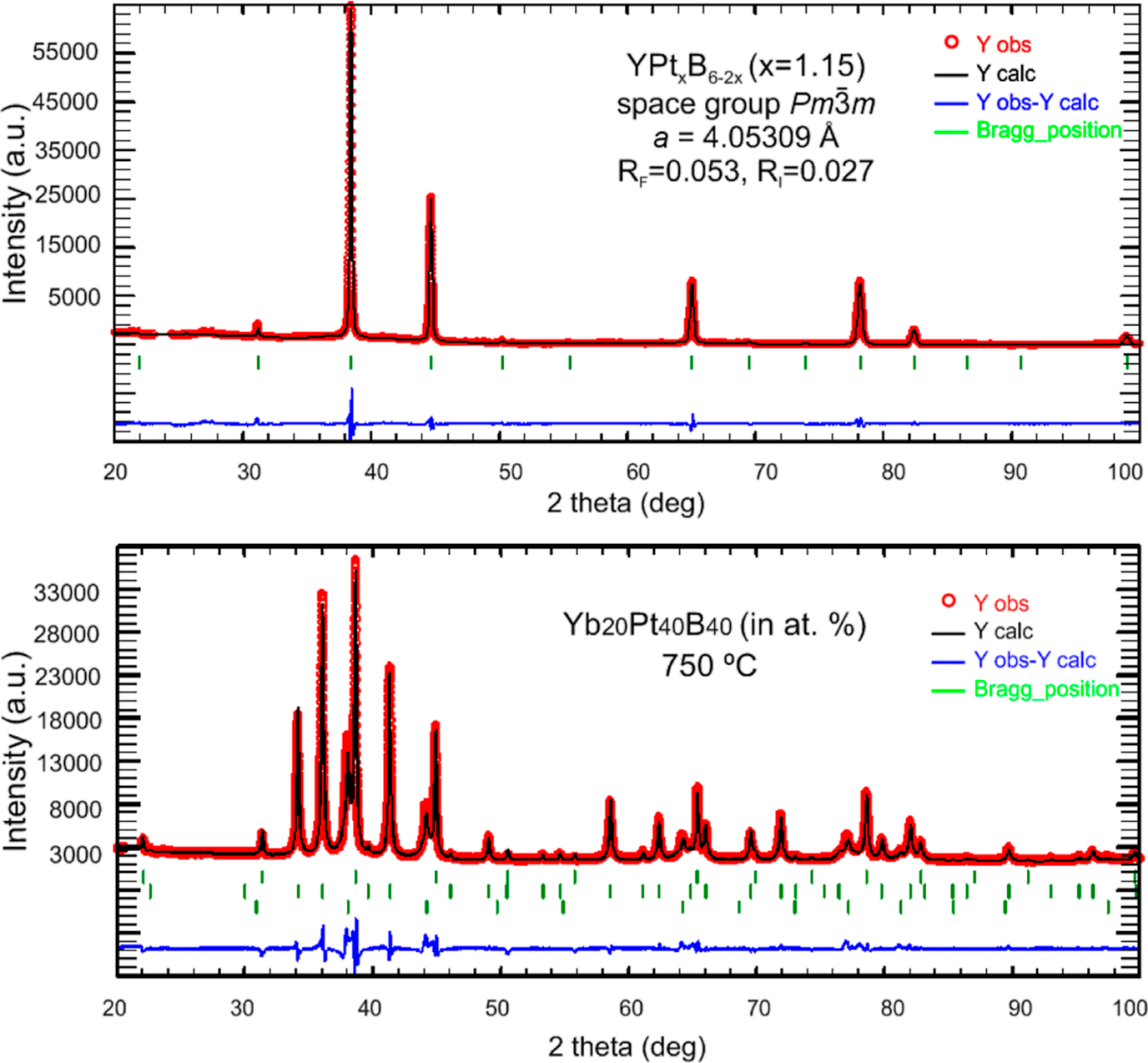
(Upper figure): X-ray powder diffraction pattern of YPt_*x*_B_6–2*x*_, *x* = 1.15 annealed at 780 °C. (lower figure): X-ray powder diffraction pattern of the Yb_20_Pt_40_B_40_ alloy (in at. %) annealed at 750 °C: upper row of *hkl* labels corresponds to YbPt_*x*_B_6–2*x*_ (space group *Pm*3̅*m*, *a* = 4.03372(5) Å), middle row stands for YbPt_2_B^66^ [space group *P*6_2_22, *a* = 5.24862(8) Å, *c* = 7.8739(1) Å], and lower row represents the phase YbPt_3_B_*x*_^67^ (space group *Pm*3̅*m*, *a* = 4.0969 Å).

All reflections collected during single crystal diffraction measurements on both crystals could be indexed with a primitive cubic unit cell (parameters are given in Table 1). No reflections, which could indicate either a modulation or changes in unit cell dimensions, were observed. The analysis of the extinction conditions indicated several possible space groups (*Pm*3̅*m*, no. 221; *P*4̅3*m*, no. 215; *P*432, no. 207; *Pm*3̅, no. 200; *P*23, no 195), of which the higher symmetric *Pm*3̅*m* was chosen to determine the structure. By using direct methods, the initial structure solutions were performed, yielding structure models with composition MPt_3_ (M = Y, Yb), and anomalously contrasting values of atomic isotropic displacement parameters. Upon refining the occupancy of Pt atom sites (approximately 40%), the residual electronic peaks of 6.20 e^–^/Å^3^ for YPt_*x*_B_6–2*x*_ and 5.64 e^–^/Å^3^ for YbPt_*x*_B_6–2*x*_ at 6f (∼0.20, 12, 12) have been revealed in the difference Fourier maps. The refinements have been significantly improved by assignment of those peaks to B atoms in both structures and constraining the overall occupation factors of Pt and B sites to 1.00. To estimate the correct compositions, the constrained occupancy parameters were refined in a separate series of least–squares cycles, yielding reasonable ADP values at the population level of 38.3% Pt/61.7% B and 44.5% Pt/55.5% B, thus delivering the formulae YPt_1.15_B_3.70_ and YbPt_1.34_B_3.33_, respectively. The final refinements with fixed occupancy parameters for Pt/B split atom sites converged to a reliability factor value of *R*_F_^2^ = 0.0284 and *R*_F_^2^ = 0.0315 exhibiting small residual electron densities for yttrium and ytterbium crystals, respectively. The crystallographic data, final atomic coordinates, and displacement parameters are presented in Table 1 and interatomic distances are listed in Table 2. To test the solution suggested in ref (32), both structures were solved and refined in the space group *Pm*3̅ (no. 200). Refinements resulted in slightly higher values of reliability factors (*R*_F_^2^ = 0.0334 in *Pm*3̅ vs *R*_F_^2^ = 0.0284 in *Pm*3̅*m* for YPt_*x*_B_6–2*x*_ and *R*_F_^2^ = 0.0360 in *Pm*3̅ vs *R*_F_^2^ = 0.0315 in *Pm*3̅*m* for YbPt_*x*_B_6–2*x*_). Moreover, the Platon’s ADDSYM tests on missed translation symmetry (based on Le Page’s MISSYM^63,64^ algorithm) incorporated in WINGX^65^ reported on potential additional 4-fold rotation axes and *m* planes suggesting the *m*3̅*m* Laue symmetry in both unit cells. Thus, the *Pm*3̅*m* space group has been chosen as the correct one for the {Y,Yb}Pt_*x*_B_6–2*x*_ crystals studied. As inferred from Rietveld refinement, powder XRD intensities collected from the polycrystalline alloys [Y_17_Pt_20_B_63_ and Yb_20_Pt_40_B_40_ (in at.%)] are in best agreements with the intensities calculated from the structural model taken from the single crystals (Figure 1).

**Table 2:** Selected Interatomic Distances (in Å) in MPt_*x*_B_6–2*x*_ in Comparison with Interatomic Distances in MB_6_ and MPt_3_ (M = Y, Yb)

YPt_*x*_B_6__–__2*x*_	YB_6_^75^	YPt_3_^76^
Y–B 2.996(6)	Y–B 3.012	Y–Pt 2.877
Y–Pt 2.8673(3)	B–B^a^ 1.630	Pt–Pt 2.877
Pt–Pt 2.8673(3)	B–B^b^ 1.746	
Pt–B 2.335(10)		
B–B^a^ 1.74(4)		
B–B^b^ 1.64(3)		

YbPt_*x*_B_6__–__2*x*_	YbB_6_^77^	YbPt_3_^76^
Yb–B 2.980(7)	Yb–B 3.049	Yb–Pt 2.853
Yb–Pt 2.8602(1)	B–B^a^ 1.669	Pt–Pt 2.853
Pt–Pt 2.8602(1)	B–B^a^ 1.753	
Pt–B 2.344(12)		
B–B^a^ 1.67(4)		
B–B^b^ 1.68(3)		

a Interoctahedral (B–B_out_) bonds.

b Intraoctahedral (B–B_in_) bonds.

### 2.3 Electronic Structure Calculations and Chemical Bonding Analysis

Band structure (electron dispersion) and density of states (eDOS) calculations were performed within the DFT framework using the Quantum ESPRESSO 6.6 package.^68^ Correlation and exchange effects of the electrons were handled utilizing the generalized gradient approximation (GGA) of Perdew, Burke, and Ernzerhof, revised for solids (PBEsol).^69^ Electron–ion interactions were treated using both fully relativistic and pseudorelativistic projector augmented wave (PAW^70,71^) potentials constructed according to the code supplied by the PSLibrary (version 1.0.0).^72^ The electron wave functions were expanded into plane waves with a kinetic energy cutoff of 77 Ry. For the charge density, a kinetic energy cutoff of 329 Ry was used. The unit cell parameters and atomic positions of YPt_3_ and YB_6_ were relaxed using the Broyden–Fletcher–Goldfarb–Shanno (BFGS) algorithm and a 14 × 14 × 14 *k*-point mesh constructed using the Monkhorst–Pack method^73^ that guarantees less than 0.02 × 2π/Å spacing between the *k*-points. The version of Quantum Espresso employed does not allow optimization of the volume of the unit cell; thus, in the case of YPt_*x*_B_6–2*x*_ modeled structures, a different procedure was used to find the most energetically favorable cell parameters. The cell parameters were varied in the vicinity of the experimental values, followed by a relaxed calculation to determine the minimal energy of every configuration. The cell parameters corresponding to the minimal energy were found using a polynomial fit. Finally, the structure was relaxed for the atomic coordinates. All calculations were performed on a 14 × 14 × 14 (type-**I** YPt_*x*_B_6–2*x*_) and 7 × 7 × 14 (type-**II** YPt_*x*_B_6–2*x*_) *k*-point mesh. The convergence threshold for self-consistent-field iteration was set at 10^–9^ eV. Density of states integrations within the irreducible wedge of the primitive Brillouin zone and electron localization function (ELF) calculations were completed on a dense *k*-point mesh (half the spacing used for the relaxation procedure). ELF distribution was analyzed and visualized using VESTA v.3.5.8 software.^74^

## 3 Results and Discussion

### 3.1 MPt_*x*_B_6–2*x*_ (M = Y, Yb): Structural Description and Analysis

**Figure 2:**
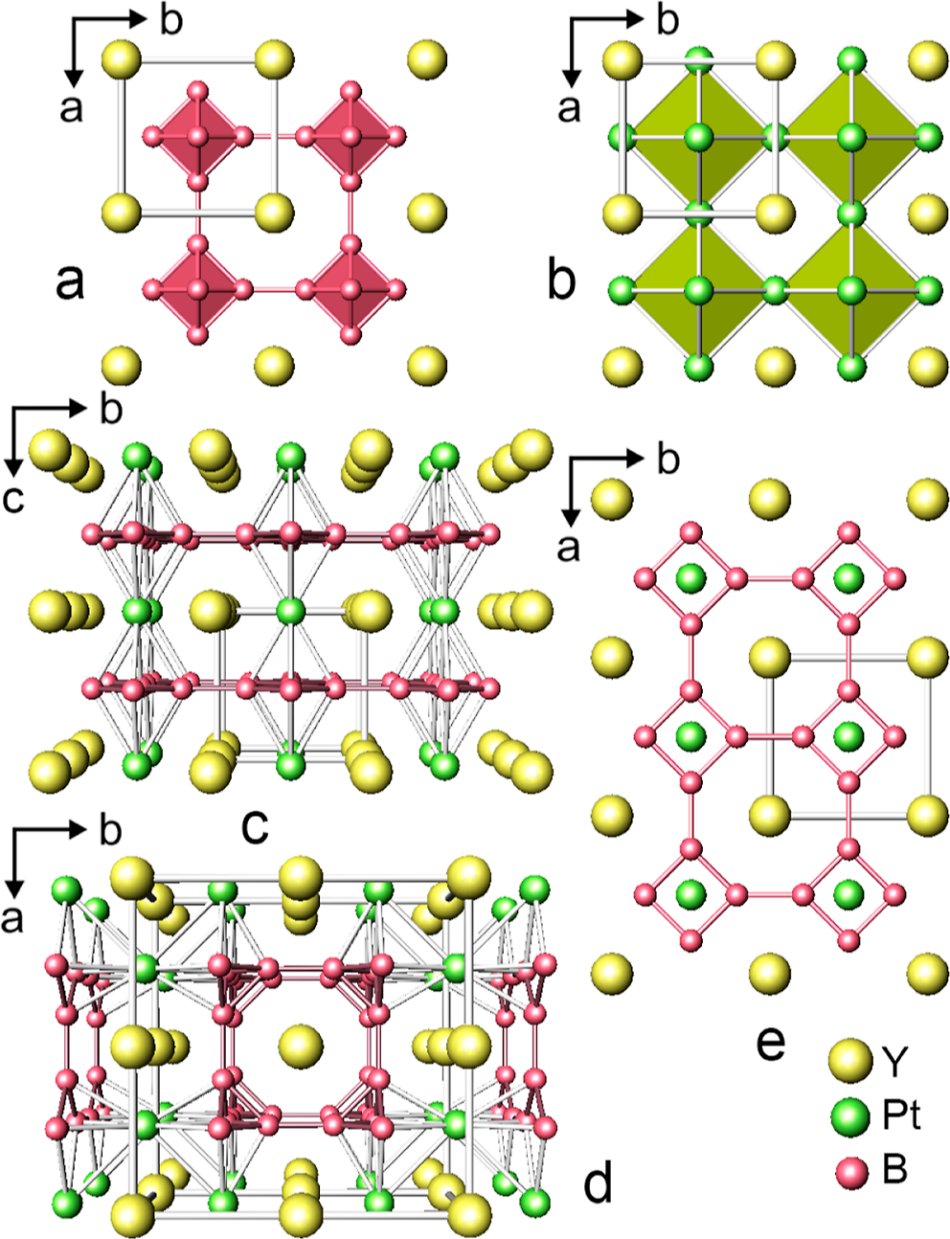
Structures of MB_6_ (CaB_6_-type; [B_6_] octahedra in red) (a) and MPt_3_ (AuCu_3_-type; [Pt_6_] octahedra in green) (b). Perspective views of type-**I** YPt_*x*_B_6–2*x*_ structure model along the *a* axis (c) and type-**II** YPt_*x*_B_6–2*x*_ structure model along the *c* axis (d). 4.8^2^ nets of B in the type-**I** YPt_*x*_B_6–2*x*_ (e). Red sticks correspond to B–B bonds, and white sticks denote Pt–Pt and Pt–B bonds. The remaining bonds are omitted.

As reflected by the refined formulae, the crystal structure of MPt_*x*_B_6–2*x*_ (M = Y, Yb) is a combination of MB_6_ (CaB_6_-type, 3D-framework of [B_6_] octahedra) (Figure 2a) and MPt_3_ (AuCu_3_-type, 3D-framework of vertice-sharing [Pt_6_] octahedra) (Figure 2b) where M atoms occupy the cube corners, while platinum and boron atoms partially occupy the face-centered position [3c (0,1/2,1/2)] and octahedral sites inside the cube [6f (*x*,1/2,1/2)], respectively. Accordingly, the M atom (in 1a atom site) is surrounded by 24 boron atoms which form the truncated cube and the coordination polyhedron for B is a tetragonal antiprism capped with a B atom [BB_5_M_4_]. At this event, when platinum is found in the 3c Wyckoff position, the coordination polyhedra of M and Pt are [MPt_12_] and [PtPt_8_M_4_] cuboctahedra, respectively. Thus, assuming random occupancy of either boron or platinum atom sites, the coordination of atoms corresponds to those found in MB_6_ and MPt_3_. At variance from general features of the RE hexaboride family (i.e., interoctahedral B–B_out_ bonds are usually shorter than intraoctahedral B–B_in_ separations),^75−78^ the B–B_in_ and B–B_out_ bonds in YbPt_*x*_B_6–2*x*_ are almost equal, while boron involved interatomic distances in YPt_*x*_B_6–2*x*_ exhibit an opposite behavior to YB_6_ (Table 2) indicating relatively weak exohedral bonds. The M–Pt, M–M, and M–B interatomic distances in the Y and Yb structures agree well with distances between atoms in binary MPt_3_ and MB_6_ (M = Y, Yb).

The situation, where XRD refinement of complex crystal structures results in split of certain atom positions but no superstructure reflections have been seen from XRD data, have been reported for a number of compounds, e.g., boron carbide,^79^ Sc_5_Rh_6_Sn_18_,^80^ Ba_7.81_Ge_40.67_Au_5.33_,^81^ Mg_2_Rh_1–*x*_B_6+2*x*_,^82^ etc. In order to resolve the partial occupation of platinum and boron sites and create ordered models suitable for quantum chemical calculations, two structural variants were developed via the group-subgroup approach accounting for the target atomic composition ratio (i.e., ∼1Y:1Pt:4B for YPt_*x*_B_6–2*x*_): type-**I**, space group *P*4/*mmm*, no. 123, *a*′ = *a*, *b*′ = *b*, *c*′ = *c*; and (ii) type-**II**, space group *P*4/*mmm*, no. 123, *a*′ = 2*a*, *b*′ = 2*b*, *c*′ = *c* (Figure 2c–e). The atomic coordinates of the two structural models of YPt_*x*_B_6–2*x*_ obtained via transformations are presented together with optimized structural parameters in Section 3.3.

In comparison to the YB_6_ structure, the type-**I** model implies the replacement of two boron atoms lying along the same axis by a single Pt atom, followed by a shift onto the plane, which is perpendicular to the mentioned axis. Platinum atoms together with yttrium atoms in such a structural array assemble a single (001) family of lattice planes (Figure 2c). The type-**II** structure model is formed by removing the two boron atoms that lie along two different axes. This leads to the placement of platinum atoms on two perpendicular [(100) and (010)] families of lattice planes in a supercell formed from four initial cubic YB_6_ unit cells (Figure 2d). Such a supercell is also tetragonal with cell parameters *a*′ = 2*a*, *b*′ = 2*b*, *c*′ = *c* and also belongs to the *P*4/*mmm* space group. In the ordered models two kinds of boron aggregations form, i.e., (i) infinite parallel 2D nets of boron octagons condensed via common edges to form the 4.8^2^ nets interlinked via yttrium and platinum atoms (Figure 2e) (type-**I**, *a*′ = *a*, *b*′ = *b*, *c*′ = *c*), and (ii) the columns of linked (via common octagonal faces) [B_24_] truncated cubes filled with Y running along the *c* axis (Figure 2d) (type-**II**, *a*′ = 2*a*, *b*′ = 2*b*, *c*′ = *c*). The isotypic ytterbium structure exhibits an increase of Pt content in the unit cell (i.e., ∼1.5Pt:3B for YbPt_*x*_B_6–2*x*_, *x* = 1.34), suggesting further disfragmentation of the octahedral boron framework. In both type-**I** and type-**II** YPt_*x*_B_6–2*x*_ models, boron atoms reside inside a boron-capped tetragonal antiprism; in contrast to YB_6_ ([BB_5_Y_4_], Figure S1b), there are three types of tetragonal antiprisms instead of one, because of a replacement of boron atoms by platinum atoms at certain atom sites of the two superstructures (Table S1 and Figures S2c and S3e,f). The antiprisms filled with boron are distorted as compared to B-filled antiprisms in YB_6_ and have edges of different lengths. Further details on atomic coordination in two structural models obtained via group-subgroup relationships are discussed in the Supporting Information.

The planar nets composed of condensed boron rings (five-, six-, and seven-membered) interleaved with metal layers are often encountered in the structures of borides with boron-to-metal ratio larger than 1.5 and have been widely discussed.^83^ Slightly puckered nets of condensed four- and seven-membered rings occur in Er_4_NiB_13_, which is a superstructure to tetraboride UB_4_ where [B_6_] octahedra interlinked via boron atoms build a three-dimensional network. The distinct motif of 4.8^2^ nets has been previously observed in the so-called “YB_2_C_2_” structural family,^84−87^ where infinite planar nets of fused [B_2_C_2_] and [B_4_C_4_] rings interleave with the layers of metal atoms. In the boron carbide structures, the metal atoms center only the [B_4_C_4_] rings, while in the structure of type-**I** YPt_*x*_B_6–2*x*_, both [B_4_] squares and [B_8_] octagons are centered by metal atoms, i.e., Pt and Y, respectively (Figure S4).

In the type-**II** YPt_*x*_B_6–2*x*_ structure model, the columns of Y filled [B_24_] cages interleave along *a* and *b* directions with metal nets composed of yttrium and platinum. The [B_24_] cage exhibits six B_8_ faces joined via common edges and triangular faces to form the truncated cube. Alternatively, the [B_24_] unit can be described as two octagonal planar boron rings, bridged via additional B atoms in a manner “one B–B chain (i.e., B_2_ unit) per two boron atoms of each ring”. The planar eight-membered boron rings, interconnected via B–B dumbbells have been found in ErNiB_4_ (space group *I*4/*mmm*)^88^ (Figure S5); this structure also features the [B_4_Ni_2_] octahedra, comparable to [B_4_Pt_2_] octahedra which form upon substitution of two boron atoms by platinum in the constructed type-**II** YPt_*x*_B_6–2*x*_ structure model. Comparative analysis showed that the unit cell of ErNiB_4_ is composed of two unit cells of type-**II** YPt_*x*_B_6–2*x*_, stacked along the *c* axis, one of which is shifted for 1/2,1/2,0 with respect to the other (Figure S6). Finally, the comparison of type-**II** YPt_*x*_B_6–2*x*_ with a recently reported family of structures, where boron exists as [B_20_] isolated units [Zn_2_Ni_21_B_20_,^89^ Ga_2_Ni_21_B_20_,^90^ and MNi_21_B_20_ (M = In, Sn)^91^ allowed to distinguish similar structural fragments (e.g., eight-membered boron rings, two-dimensional infinite planar nets formed by intercrossed chains of metal atoms) (Figure S7). The analysis described above strongly supports the actuality of the constructed structural models.

### 3.2 Electron Localization Function

To gain further insights into the chemical bonding nature in the new structure, we calculated the electron localization function (ELF) for the YPt_*x*_B_6–2*x*_ structure, which provides a description of the bond type between atoms. The ELF distributions have also been calculated and visualized for the constituent building fragments of YPt_*x*_B_6–2*x*_, the YB_6_ and YPt_3_ structures.

#### 3.2.1 YB_6_

**Figure 3:**
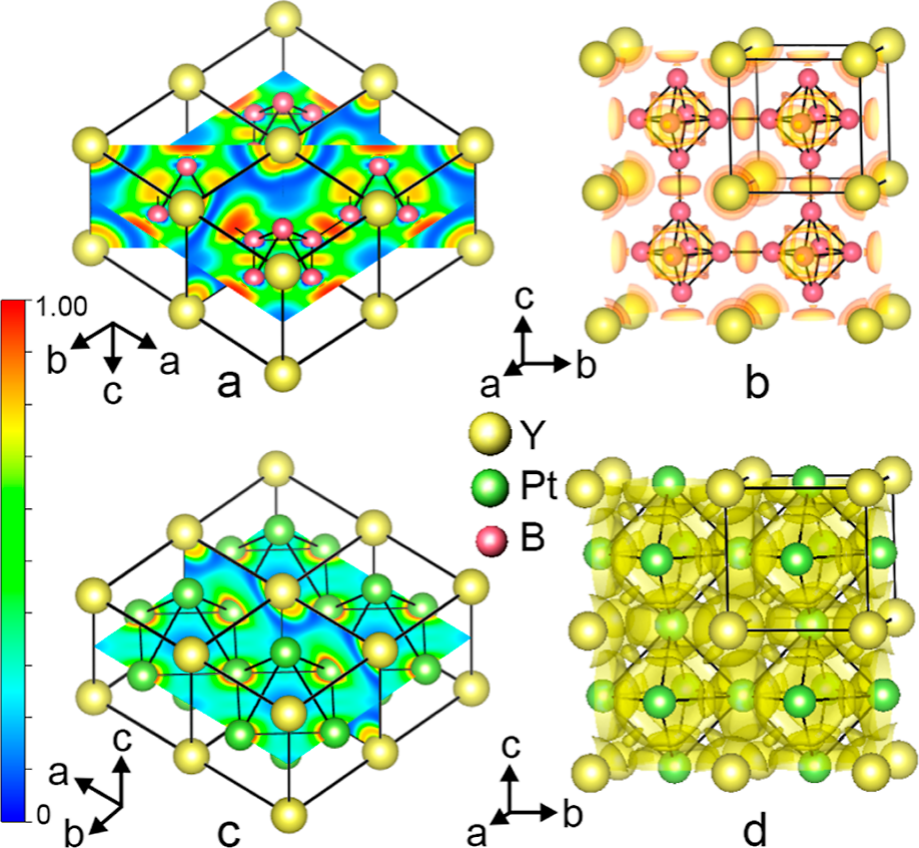
Sections of calculated electron localization function within the (100), (110), and (002) lattice planes (a) and electron localization at 0.8 isosurface level (b) within four unit cells in YB_6_. Sections of calculated electron localization function within the (100) and (002) lattice planes (c) and electron localization at the 0.3 isosurface level (d) within four unit cells in YPt_3_.

In the structure of cubic hexaboride YB_6_ with *Pm*3̅*m* symmetry, the [B_6_] octahedron is surrounded by eight nearest neighbor yttrium sites defining a cube. The [B_6_] octahedra interlink to form a three-dimensional framework; the interoctahedral B–B_out_ bonds are shorter than the intraoctahedral ones (B–B_in_) (Table 2). The calculations definitely showed the covalent bonding within the octahedral boron framework with a larger ELF value for interoctahedral B–B_out_ bonds, i.e., 0.95 versus 0.81 for intraoctahedral B–B_in_ bonds (Figure 3a). The maxima of ELF, visualized by the isosurface (at 0.8 level) (Figure 3b) are located at the [B_6_]–[B_6_] bond midpoints and above the octahedral triangular faces and represent a typical picture for boron bonding in hexaborides.^45,78,92−95^ Besides those, another ELF maximum is found in the (110) plane around the Y atom site, further followed by the almost empty region along the Y–B interatomic line (blue area, ELF value less than 0.07); the empty regions in the ELF map denote the transfer of electrons from yttrium to boron, indicating the dominating ionic character of this interaction. In accordance with a substantial charge transfer from yttrium to the boron framework, the analysis of Bader charges within the quantum theory of atoms in molecules (QTAIM) approach^96,97^ (Table 3) reveals a positive charge of +1.842*e*^–^ for yttrium and −0.307*e*^–^ for boron in excellent agreement with literature data.^92−95^

**Table 3:** Calculated Bader Charges of Y, Pt, and B Atoms in Four Considered Phases

phase	space group	atom	charge value (*e*^–^)
YB_6_	*Pm*3̅*m*	Y	+1.842
		B	–0.307
YPt_3_		Y	+1.669
		Pt	–0.556
type-**I** YPt_*x*_B_6__–__2*x*_	*P*4/*mmm*	Y in 1a (0,0,0)	+1.817
		Pt in 1c (1/2,1/2,0)	–0.745
		B in 4o (*x*,1/2,1/2)	–0.268
type-**II** YPt_*x*_B_6__–__2*x*_	*P*4/*mmm*	Y1 in 1a (0,0,0)	+1.760
		Y2 in 2f (0,1/2,0)	+1.819
		Y3 in 1c (1/2,1/2,0)	+1.825
		Pt1 in 4m (*x*,0,1/2)	–0.851
		B1 in 8r (*x*,*x*,*z*)	–0.215
		B2 in 8q (*x*,*y*,1/2)	–0.261

#### 3.2.2 YPt_3_

In binary YPt_3_ with AuCu_3_-structure (*Pm*3̅*m* space group), the Y atoms occupy the corners of the cube, and the Pt atoms occupy the cube faces. The difference in electronegativities of yttrium and platinum implies that Pt acquires some electrons from Y atoms resulting in an appearance of Y–Pt ionic bonds. Consistently, the calculated ELF distribution map for the (100) plane reveals electron localization domains (ELF = 0.8) next to the Y atom site and a low ELF value of 0.12 away from these regions (Figure 3c). The transfer of charge density from Y, which centers the [Pt_12_] cuboctahedron, to Pt atoms is also evident from the analysis of Bader charges (Table 3); the calculated values for Y and Pt agree well with the previous theoretical data for YPt_3_.^98^ The ELF distribution between adjacent Pt–Pt atoms forming the Pt_6_ octahedron is more uniform, implying its metallic character. Similar to related structures with Sc, Zr, and Hf,^98,99^ the ELF distribution in the (002) plane features the electron localization domains (ELF = 0.30), corresponding to weak pairwise Pt–Pt interactions (Figure 3c,d).

#### 3.2.3 YPt_*x*_B_6–2*x*_ Structure (*x* = 1.15)

**Figure 4:**
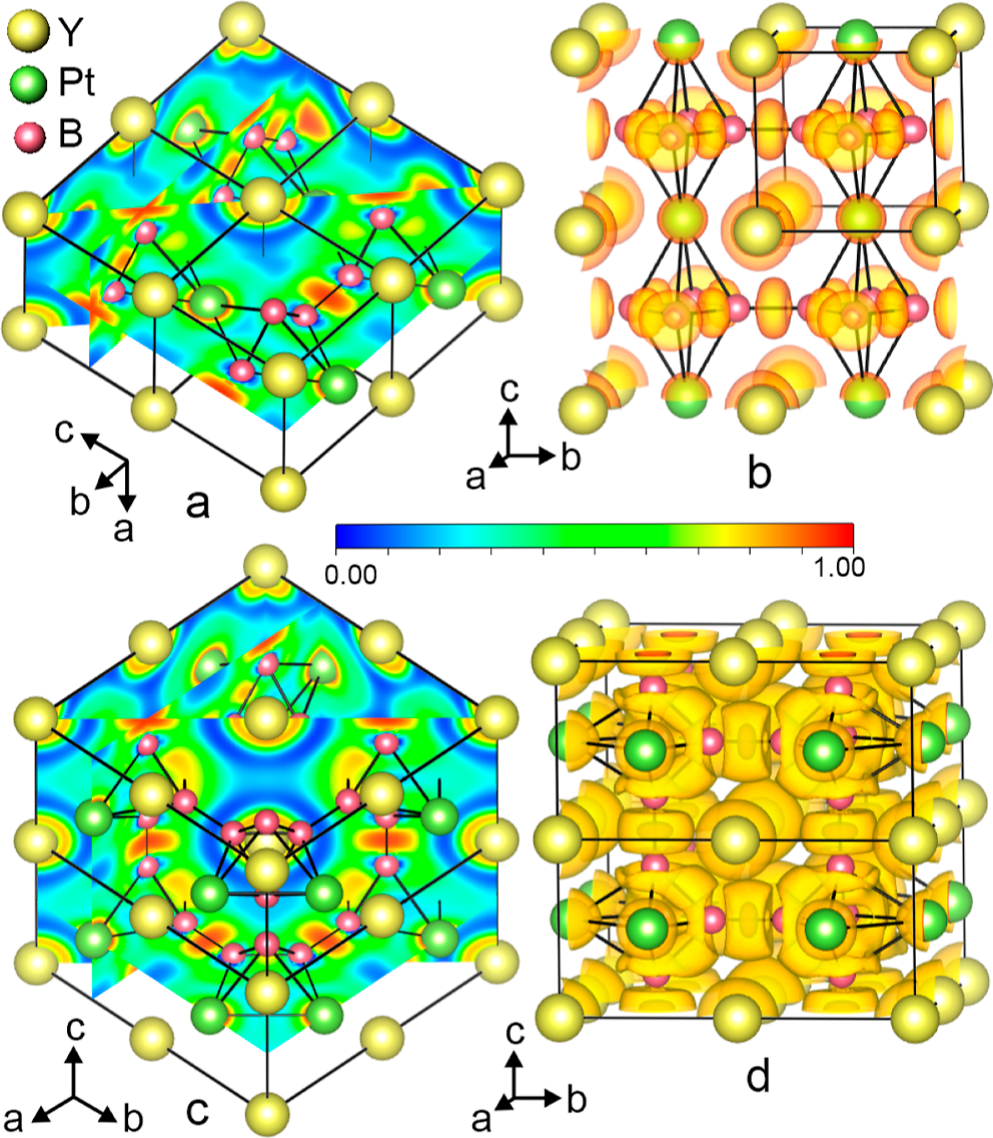
(a) Sections of calculated electron localization function in the type-**I** YPt_*x*_B_6–2*x*_ structure within the planes: (001) visualizing Y–Pt interaction; (0–10) bearing solely Y atoms, (002) showing B–B contacts within the eight- and four-membered rings; [0(−1)1] demonstrating Y–B bonding; and (200) presenting ELF distribution for B–B in B_4_ squares and Pt–B contacts. Four unit cells are drawn. Detailed visualization of the ELF distribution within the lattice planes is given in Figure S8. (b) Electron localization at 0.75 isosurface level in type-**I** YPt_*x*_B_6–2*x*_. (c) Sections of calculated electron localization function in the type-**II** YPt_*x*_B_6–2*x*_ within the planes: (100) and (010), showing Y–Pt interactions; (040), revealing Pt–B and B–B_out_ (interoctahedral) interactions; (002) presenting Pt–Pt, Pt–B, and B–B_out_ (interoctahedral) interactions; (110) intersecting the B_3_ faces of [B_4_Pt_2_] octahedra and visualizing the B–B_in_ (intraoctahedral) bonding as well as Y–B and B–B_out_ (interoctahedral) interactions. Two unit cells along *c* are shown. Detailed visualization of the ELF distribution within the lattice planes are given in Figure S9. (d) Electron localization function at 0.60 and 0.90 isosurface level (orange and brown color, respectively) in the type-**II** YPt_*x*_B_6–2*x*_.

To derive the electronic structure and to analyze the bonding of disordered YPt_*x*_B_6–2*x*_ (*x* = 1.15), the cubic symmetry *Pm*3̅*m* was reduced to tetragonal *P*4/*mmm*. Electron localization functions, plotted in the most significant crystallographic planes for the type-**I** (*a*′ = *a*, *b*′ = *b*, *c*′ = *c*) and type-**II** (*a*′ = 2*a*, *b*′ = 2*b*, *c*′ = *c*) structure models are shown in Figures 4, S8, and S9.

In the type-**I** model, the layers containing Y and Pt interleave with the layers composed of 4.8^2^ boron nets along the *z* direction (Figure 4a) and correspond structurally to the substructures of YPt_3_ (Figures 3c and S8a) and YB_6_ (Figures 3a, and S8c,b), respectively. Consequently, the Y species are well separated from the neighboring Pt and B by regions with small ELF values (Figure S8a,d) evidencing the cationic character of Y in good agreement with electronegativity values and Bader charges (Table 3). Within the planar boron network, the covalent bond between the [B_4_] squared groups is only slightly stronger than the B–B interaction within the [B_4_] unit (ELF = 0.95 and ELF = 0.89, respectively) (Figure S8c) exhibiting good agreement with bonding features of earlier described borides with planar boron networks.^82,100^ The third type of bonding in the type-**I** YPt_*x*_B_6–2*x*_ structure model occurs from Pt–B interaction; the ELF along Pt–B interatomic lines ranges in values within 0.55–0.25 implying metallic character of this interaction (Figure 4a and S8f).

The analysis of the ELF distribution in the type-**II** YPt_*x*_B_6–2*x*_ supercell (Figures 4c,d and S9) revealed predominantly ionic bonding for the Y–Pt and Y–B interactions. Similarly to YPt_3_ (Figure 3c,d), a small domain of ELF (0.31) is observed between two neighboring Pt atoms, indicating weak pairwise interactions (Figure 4d). For B–B bonds, ELF maxima are observed along the longer 1.74 Å B–B contacts connecting the [B_4_Pt_2_] octahedra (max value ∼0.950), suggesting 2c–2e bonds (Figure S9c). The interoctahedral B–B_out_ distances in this structure are more comparable with the distances B–B_in_ in YB_6_; however, elongated B–B distances are a common feature in intermetallic borides, e.g., ScRu_2_B_3_,^101^ MNi_21_B_20_ (M = In, Sn),^91^ ErNiB_4_,^88^ Ni_12_ZnB_8–*x*_,^89^ Ni_21_Zn_2_B_20_,^89^ Ni_3_ZnB_2_,^89^ etc. The maxima of ELF are also present within the faces of the [B_4_Pt_2_] octahedra (Figure S9e,f) ranging from 0.82 (at/slightly above the B_3_ faces) to 0.60 (at BPt_2_ faces) indicating B–B and B–Pt covalent bonding (Figure 4d). It should be noted that the electron localization maxima between transition metal and boron atoms (evidencing M-B covalent bonding) were earlier observed in the compounds Mg_8_Rh_4_B,^102^ Mg_1–*x*_RhB,^103^ MNi_21_B_20_ (M = In, Sn),^91^ and Ni_3_ZnB_2_.^104^

### 3.3 Density Functional Theory Calculations

To fully understand the impact of platinum on the electronic DOS of YB_6_, calculations for YB_6_ and YPt_3_ have been performed with both fully relativistic and scalar relativistic potentials. Results of the structure relaxation of these two compounds are summarized in Table 4.

**Table 4:** Structural Characteristics of YB_6_ and YPt_3_

	YB_6_^a^	YB_6_^105^	YPt_3_^a^	YPt_3_^105^
*a*, Å	4.0781 (non SOC)	4.1010–4.1560	4.0585 (non SOC)	4.069–4.075
	4.0782 (SOC)		4.0570 (SOC)	
Y in 1a (0,0,0)	0,0,0	0,0,0	0,0,0	0,0,0
Pt in 3c (0,1/2,1/2)			0,1/2,1/2	0,1/2,1/2
B in 6f (*x*,1/2,1/2)	0.1987,1/2,1/2 (non SOC)	0.1988		
	0.1987,1/2,1/2 (SOC)			

a Current work.

Both sets of potentials provide results in a very good agreement to literature data and are slightly lower than the experimental ones, as expected for the ground state. For YB_6_ the cell parameters and boron atomic coordinates obtained with and without spin–orbit interactions match up to the fourth digit, hinting at a negligible influence of SOC in this system. For YPt_3_, on the other hand, judging from the cell parameter values, the SOC is rather small but not negligible.

**Figure 5:**
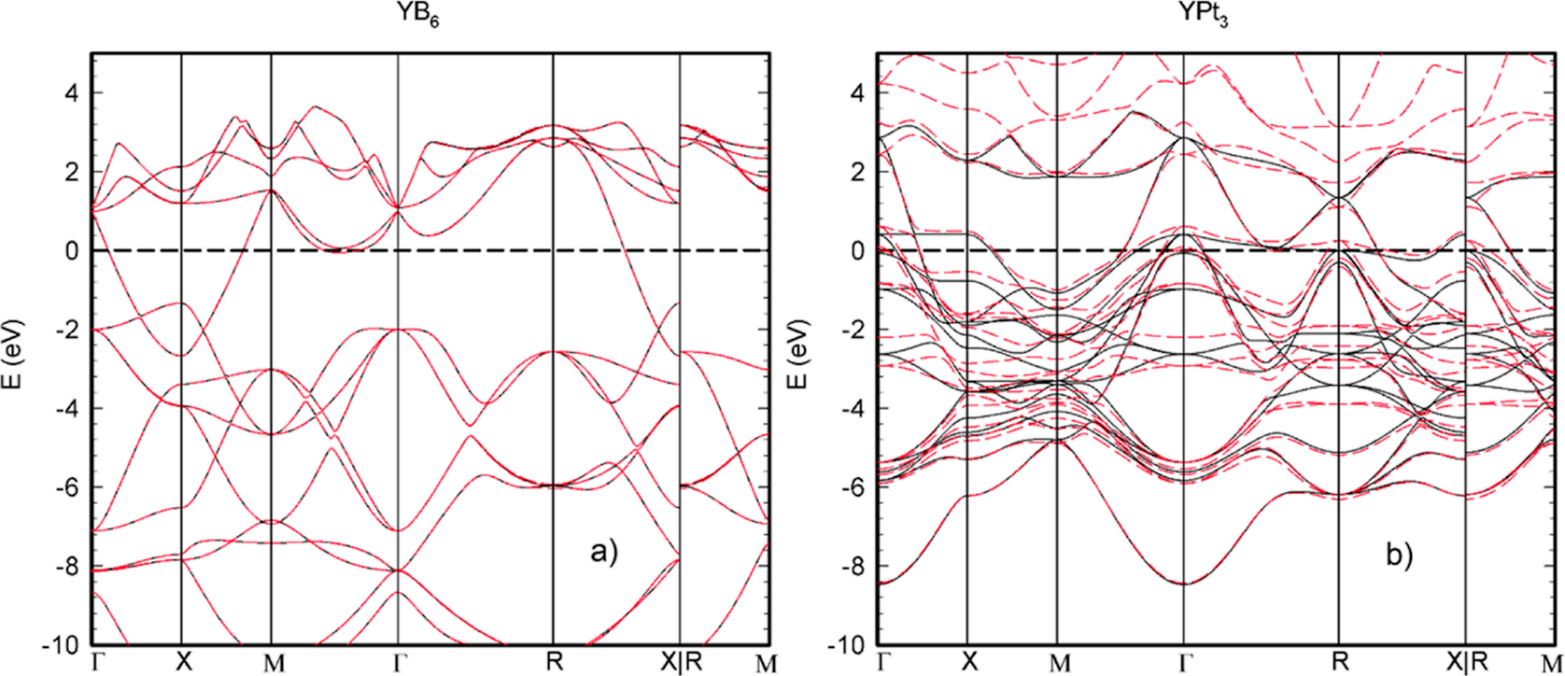
Band structure of YB_6_ (a) and YPt_3_ (b) along high symmetry directions. Solid and dashed lines correspond to results of collinear and noncollinear calculations, respectively.

To further check the correctness of the potentials used in our study, the band structures of YB_6_ and YPt_3_ along the high symmetry direction have been calculated and are presented in Figure 5 for both the relativistic and nonrelativistic case.

No spin–orbit-induced splitting was observed for the bands of YB_6_ with the potentials applied. The overall shape of the band structure of YB_6_ is in agreement with the results previously reported for the calculations performed in an LDA^37^ and GGA^7^ framework, however, differs from the results of B3LYP calculations.^106^

For YPt_3_, the SOC-driven splitting of bands is noticeable both below and above the Fermi level. The results of the non-SOC calculations for the YPt_3_ system are in good agreement with the results of previously performed studies using the GGA approximation^107,108^ further proving the appropriate choice of the potentials in use. For the calculations performed by applying full-relativistic potentials, the band structure of YPt_3_ shows decent splitting in the region around the Fermi level.

**Figure 6:**
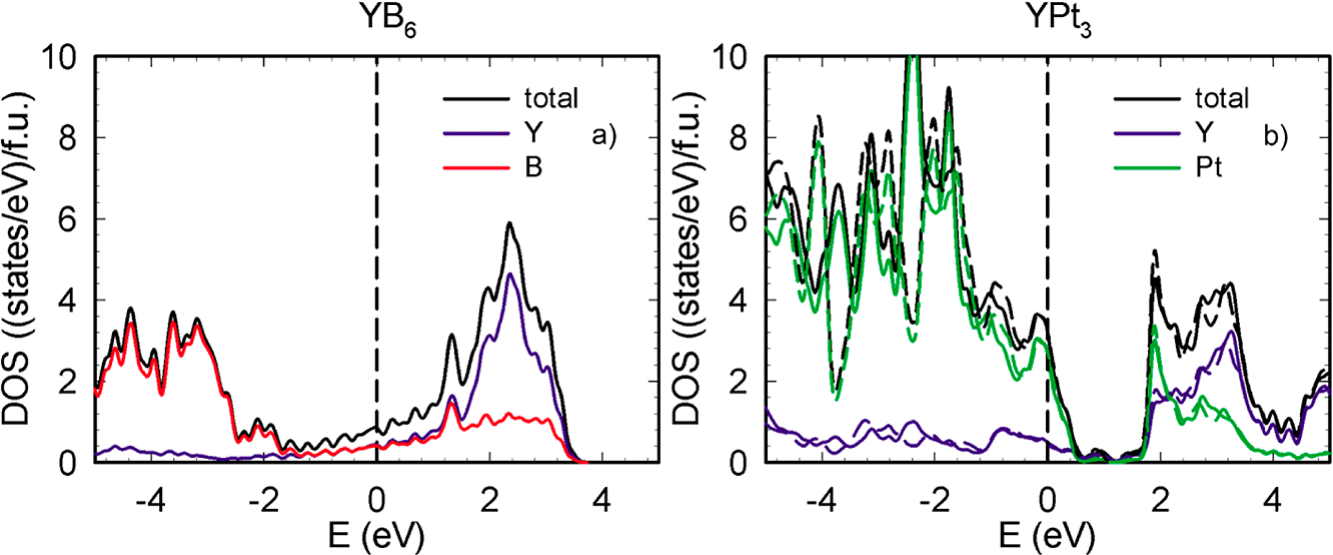
Electron density of states of YB_6_ (a) and YPt_3_ (b). Dashed and solid lines correspond to results of SOC and non-SOC calculations, respectively.

The density of states of YB_6_ and YPt_3_ are presented in Figure 6a,b, respectively. The density of states of YB_6_ is characterized by a strong hybridization of Y d- and B p-states around the Fermi level revealing 0.86 eV^–1^/f.u. at *E*_F_. The valence band of YB_6_ is dominated by boron p-states. YPt_3_ exhibits a pseudo gap between 0.5 and 2 eV in its density of states, accompanied by 3.2 eV^–1^/f.u. at the Fermi level. Pt d-states dominate the DOS of YPt_3_ at the Fermi level and in the valence band. In both compounds, the conduction band above 2 eV is dominated by yttrium d-states. The results of the calculations of partial DOS for individual atoms of YPt_3_ and YB_6_ can be found in Supporting Information, Figure S10.

To shed light on the electronic properties of YPt_*x*_B_6–2*x*_, the electronic band structure and density of states have been calculated for two structural variants, type-**I** YPt_*x*_B_6–2*x*_ and type-**II** YPt_*x*_B_6–2*x*_.

Structural parameters of these two structures were optimized using the procedure described above considering *a*′ = *a*, *b*′ = *b*, and *c*′ = *c* for type-**I** YPt_*x*_B_6–2*x*_ and *a*′ = 2*a*, *b*′ = 2*b*, and *c*′ = *c* for type-**I** YPt_*x*_B_6–2*x*_. The total energy of type-**I** and type-II YPt_*x*_B_6–2*x*_ versus cell parameter for calculations, performed both with and without spin–orbit coupling, can be found in Figure S11. The results of structure relaxation are summarized in Table 5 together with the experimental data on YPt_*x*_B_6–2*x*_.

**Table 5:** Structural Characteristics of Type-**I** and Type-**II** YPt_*x*_B_6–2*x*_

	type-**I** YPt_*x*_B_6__–__2*x*_	type-**II** YPt_*x*_B_6__–__2*x*_	type-**I** YPt_*x*_B_6__–__2*x*_ (experimental)^a^	type-**II** YPt_*x*_B_6__–__2*x*_ (experimental)^a^
cell parameters, Å	*a* = 4.0705 (non SOC)	*a* = 8.1244 (non SOC)	*a* = 4.0550	*a* = 8.1100
	*a* = 4.0692 (SOC)	*a* = 8.1238 (SOC)	*c* = 4.0550	*c* = 4.0550
		*c* = 4.0622 (non SOC)		
		*c* = 4.0619 (SOC)		
Y1	1a (0,0,0)	1a (0,0,0)	1a (0,0,0)	1a (0,0,0)
Y2		2f (0,1/2,0)		2f (0,1/2,0)
Y3		1c (1/2,1/2,0)		1c (1/2,1/2,0)
Pt1	1c (1/2,1/2,0)	4m (x,0,1/2)	1c (1/2,1/2,0)	4m (*x*,0,1/2)
		*x* = 0.2487		*x* = 0.2500
B1	4o (*x*,1/2,1/2)	8r (*x*,*x*,*z*)	4o (*x*,1/2,1/2)	8r (*x*,*x*,*z*)
	*x* = 0.1985 (non SOC)	*x* = 0.2391, *z* = 0.1997 (non SOC)	*x* = 0.215	*x* = 0.2500, *z* = 0.2150 (*x*,*y*,1/2)
	*x* = 0.1986 (SOC)-	*x* = 0.2388, *z* = 0.1997 (SOC)		
B2		8q (*x*,*y*,1/2)		8*q* (*x*,*y*,1/2)
		*x* = 0.6047, *y* = 0.2387 (non SOC)		*x* = 0.6075, *y* = 0.2500
		*x* = 0.6048, *y* = 0.2385 (SOC)		

a Transformed from space group *Pm*3̅*m*.

As can be seen from the optimization results, the cell parameters *a* and *c* for type-**I** and type-**II** YPt_*x*_B_6–2*x*_, respectively, differ by less than 0.2% both for the SOC and non-SOC calculations, hinting at the high possibility of formation of domains with a mixed structure.

Compared to the experimental data, the cell parameters of both structural variants of YPt_*x*_B_6–2*x*_ differ by less than 0.5% with values for type-**II** YPt_*x*_B_6–2*x*_ being slightly below the corresponding values for type-**I** YPt_*x*_B_6–2*x*_.

**Figure 7:**
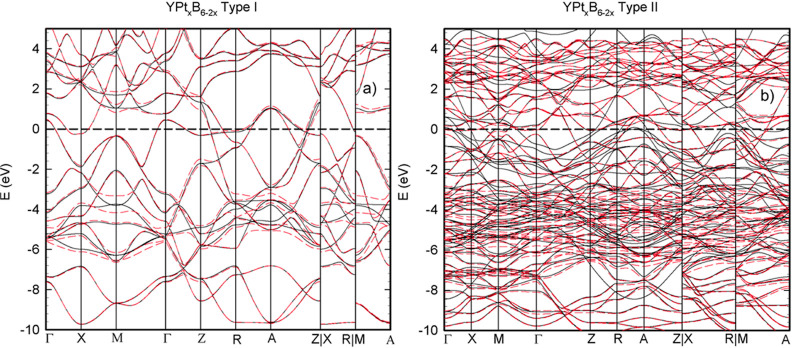
Band structure of type-**I** YPt_*x*_B_6–2*x*_ (a) and type-**II** YPt_*x*_B_6–2*x*_ (b) along the high symmetry direction. Solid and dashed lines correspond to results of calculations without and with SOC, respectively.

To analyze the electronic properties of YPt_*x*_B_6–2*x*_, the electronic band structure has been calculated for type-**I** and type-**II** YPt_*x*_B_6–2*x*_ and is presented in Figure 7. The systems have electron bands that cross the Fermi level, thus implying metallic behavior for both structures. The influence of spin–orbit interactions in the region around the Fermi level in these systems is stronger, compared to YB_6_; however, SOC mostly influences the valence band region.

**Figure 8:**
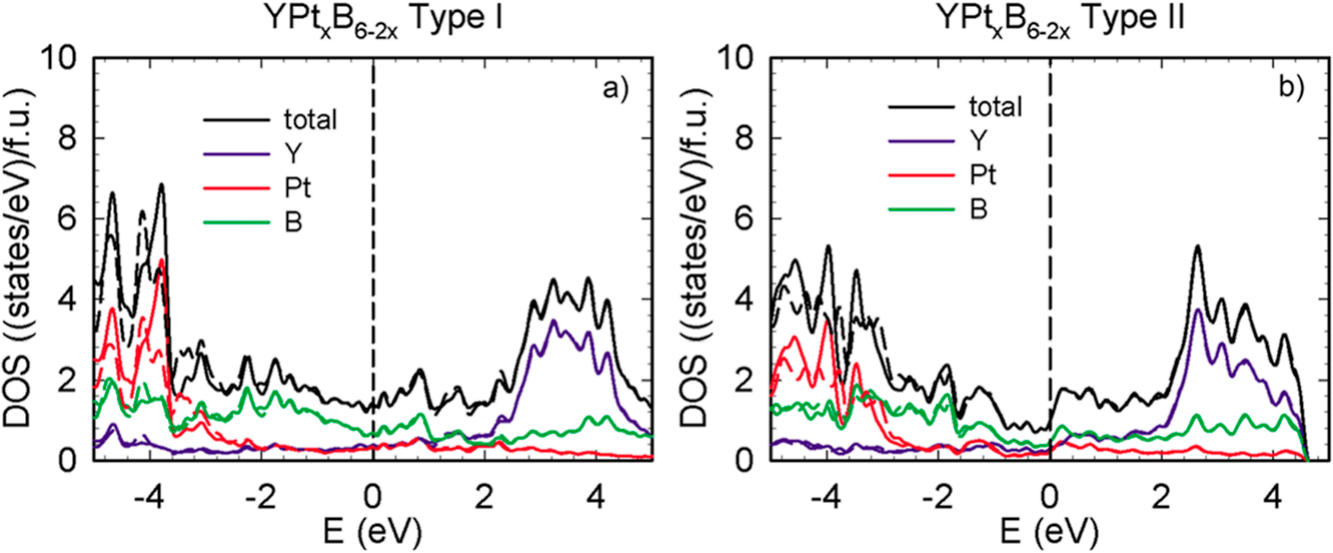
Electron density of states for type-**I** YPt_*x*_B_6–2*x*_ (a) and type-**II** YPt_*x*_B_6–2*x*_ (b). Solid and dashed lines correspond to results of calculations without and with SOC, respectively.

The electron density of states calculated for these two systems is presented in Figure 8. At the Fermi level, type-**I** YPt_*x*_B_6–2*x*_ exhibits a density of states value of 1.4 eV^–1^/f.u., while for type-**II** YPt_*x*_B_6–2*x*_, this value is smaller, around 1 eV^–1^/f.u. The values of the DOS at the Fermi energy and the specific heat constant γ for types-**II** YPt_*x*_B_6–2*x*_ and type-**I** YPt_*x*_B_6–2*x*_ together with the corresponding values for YPt_3_ and YB_6_ are summarized in Table 6.

**Table 6:** DOS (eV^–1^) at Fermi Energy and γ (J mol^–1^ K^–2^) Values per Formula Unit (f.u.) and per Atom

	DOS/f.u.	γ/f.u., mJ mol^–^^1^ K^–^^2^	γ/atom, mJ mol^–^^1^ K^–^^2^
YB_6_	0.86	2.03	0.22
YPt_3_	3.2	7.54	1.89
type-**I** YPt_*x*_B_6__–__2*x*_	1.4	3.30	0.66
type-**II** YPt_*x*_B_6__–__2*x*_	1	2.36	0.47

Unlike YPt_3_, neither compound exhibits a pronounced pseudogap in the vicinity of the Fermi level. Similar to the case for YB_6_ and YPt_3,_ the conduction band of both structural variants above 2 eV is dominated by Y d-states. As the energy frame moves below 2 eV, the total density of states for both types decreases and the influence of boron states become more prominent. At the Fermi level, the density of states of type-**I** YPt_*x*_B_6–2*x*_ consists of boron states (50%) as well as of Y and Pt states (25% each); type-**II** YPt_*x*_B_6–2*x*_ is formed by 50% boron states, too, with Y and Pt contributing to the remaining rest. In the valence band below the Fermi level, the density of states is dominated by boron p-states down to −3 eV, where the Pt d-states start to dominate.

Spin orbit coupling influences mainly the d-states of platinum in both compounds, and thus, the total DOS calculated for both models is modified in the region around −4 eV.

The results of the calculations of partial densities of states for individual atoms for both models for the collinear and noncollinear case can be found in Figures S12–S14 in the Supporting Information.

### 3.4 Y–Pt–B Ternary Phase Diagram in the Relevant Concentration Area

**Figure 9:**
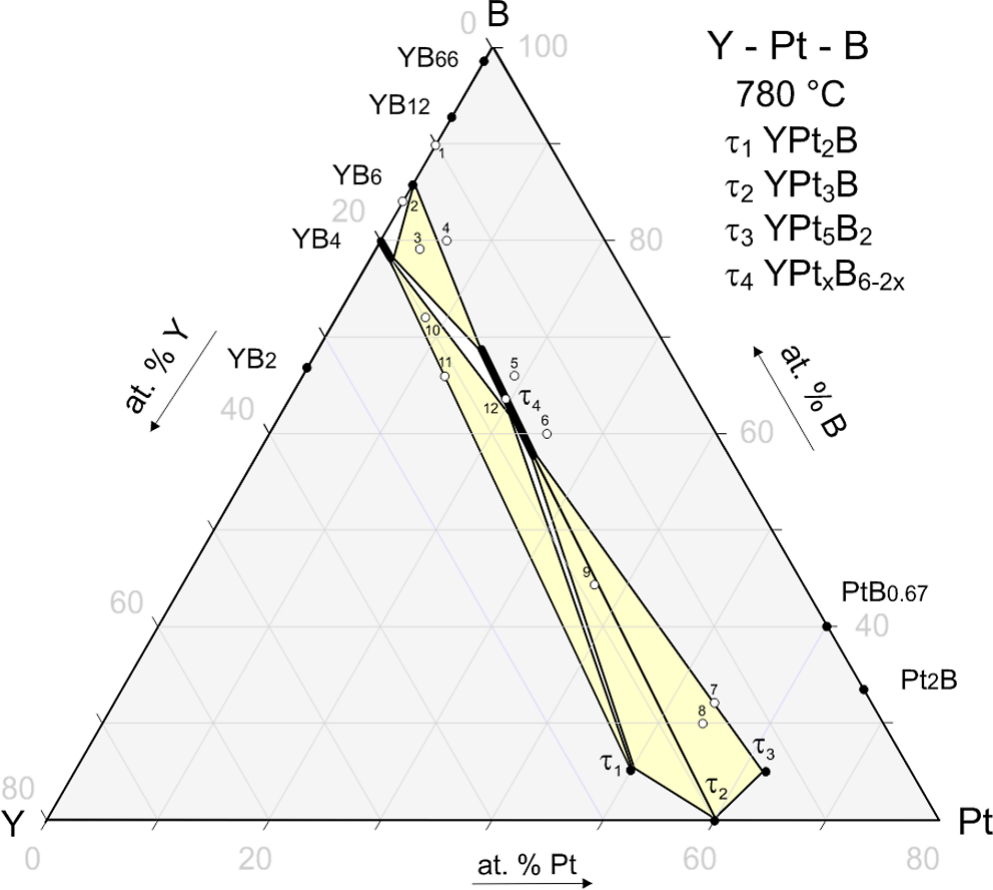
Phase relations in the 780 °C isothermal section of the Y–Pt–B system. Open circles represent the sample location. Black dots and fields denote compounds or single-phase homogeneity regions.

With respect to our strong interest in proper phase equilibria for the ternary RE–Pt–B system, a closer inspection of phase relations in the vicinity of the compound YPt_*x*_B_6–2*x*_ (*x* = 1.15) also became a subject of our study. In the current work, we observed and identified three ternary compounds, existing in equilibrium at 780 °C with YPt_*x*_B_6–2*x*_ (*x* = 1.15) (Figure 9); the crystal structures of these phases were evaluated from powder XRD data applying Rietveld refinement. YPt_2_B^54^ and YPt_3_B form isotypic structures with CePt_2_B (space group *P*6_2_22)^55^ and CePt_3_B (space group *P*4*mm*),^109^ respectively; YPt_5_B_2_ is found to be isostructural to recently reported YbPt_5_B_2_ (space group *C*2/*m*)^110^ (powder XRD, Table S2 and Figures S15 and S16).

The composition of the new phase YPt_*x*_B_6–2*x*_, as refined from single-crystal XRD data, is close to the composition of the so-called “21 K superconducting phase” Th_1–*y*_Pd_*x*_B_6–2*x*_ (*x* = 0.65, 0 ≤ *y* ≤ 0.22), which was described by Zandbergen et al. in 1995 from high-resolution electron microscopy^48^ and which was recently reported to be the end point of the Th(B,Pd)_6_ solid solution, terminating at 9 at. % Pd at 950 °C.^52^ In contrast to thorium palladium boride, YPt_*x*_B_6–2*x*_ at 780 °C is a free-standing ternary compound according to EPMA and powder XRD (Figures 9, S15–S17, Table S2). This compound forms two phase equilibria with three ternary phases YPt_5_B_2_, YPt_3_B, and YPt_2_B in the Pt-rich part of the ternary phase diagram at 800 °C. In the B-rich part, the sample no.3-Y_17_Pt_4_B_79_ (in at. %) documents the three-phase equilibrium YB_4_ + YB_6_ + YPt_*x*_B_6–2*x*_, while the sample no. 4-Y_14_Pt_6_B_80_ (in at. %) confirms the tie-line YPt_*x*_B_6–2*x*_ + YB_6_ and, additionally, reveals a third phase, obviously rich in boron. Similarly, sample 5-Y_15_Pt_19_B_66_ (in at. %) documents the tie-line YPt_*x*_B_6–2*x*_ + YPt_5_B_2_ as well as displays the pattern of weak reflections, which does not conform the reported binary and ternary compounds. In the Pt-rich region, the results of Rietveld refinement and EPMA unambiguously derived three-phase equilibria: YPt_*x*_B_6–2*x*_ + YPt_5_B_2_ + YPt_3_B, YPt_*x*_B_6–2*x*_ + YPt_3_B + YPt_2_B, and YPt_*x*_B_6–2*x*_ + YPt_2_B + YB_4_. Based on the obvious potential of transition metals to incorporate into the boron atom framework,^27,28,34^ further studies on the boron-rich part of the Y–Pt–B phase diagram, with emphasis on crystal structure and properties of ternary higher borides are in progress.^111^ No significant solubility of Pt has been observed in binary yttrium borides, pertinent to the concentration areas of the current study at 780 °C from powder XRD data and EPMA results. YB_4_ dissolves up to 3 at. % Pt according to EPMA EDX analyses; the lattice parameters of YB_4_(Pt) in ternary alloys slightly increase as compared to lattice parameters refined from the binary alloy. The lattice parameters of YB_6_ vary from 4.10030(4) and 4.10332(5) Å for Y-rich and B-rich compositions, as determined from binary alloys no. 2-Y_16_B_84_ (in at. %) and no. 1-Y_10_B_90_ (in at. %), respectively, annealed at 780 °C (Table S2). Only slight deviations for YB_6_ lattice parameters have been found in ternary alloys (i.e., 4.0962–4.0966 Å), indicating a very small solubility of Pt in binary YB_6_, in contrast to a significant difference between lattice parameters of YPt_*x*_B_6–2*x*_ in B-poor and B-rich ternary alloys, i.e., 4.0502–4.06772 Å, respectively. These variations of lattice parameters of YPt_*x*_B_6–2*x*_ indicate the existence of a ternary homogeneity region; the solid solution extends to the limits at 0.90 ≤ *x* ≤ 1.40 as inferred from the EPMA ratio.

## 4 Conclusions

A new boron-rich compound, YPt_*x*_B_6–2*x*_ (*x* = 1.15), was obtained from arc-melted specimens, annealed at 780 °C. Its crystal structure was determined from single-crystal XRD data. The crystal structure of YPt_*x*_B_6–2*x*_ (*x* = 1.15) is derived from the fragments of YB_6_ and YPt_3_, in which YB_6_ contains [B_8_] rings and Y, and the YPt_3_ fragment contains Y/Pt layers. YbPt_*x*_B_6–2*x*_ (*x* = 1.34) was found to be isotypic from single-crystal XRD data. Structural transformation in the framework of the group-subgroup approach provided two structural variants of the disordered structure: type-**I** structure (space group *P*4/*mmm*, *a*′ = *a*, *b*′ = *b*, *c*′ = *c*) and type-**II** structure (space group *P*4/*mmm*, *a*′ = 2*a*, *b*′ = 2*b*, *c*′ = *c*). This methodology enabled structural and bonding analyses and electronic structure calculations. Thus, the type-**I** structure model yields planar 4.8^2^ nets, composed of boron atoms which, to the best of our knowledge, have not been found up to now in boride systems; both four- and eight-membered rings are centered by platinum and yttrium, respectively. The 2c–2e boron–boron interaction exists in the boron network, in good agreement with the literature data on boron bonding in related boride structures exhibiting layers of boron; the analysis of Bader charges showed that similar to YPt_3_ (also calculated within the present study), platinum acquires some charge from yttrium and does not form covalent bonds with boron in the layered type-**I** YPt_*x*_B_6–2*x*_. The most impressive feature of the type-**II** YPt_*x*_B_6–2*x*_ structure model are the [B_4_Pt_2_] octahedra formed upon replacement of two neighboring boron atoms by platinum in the [B_6_] octahedra of YB_6_. These structural units form also in the ErNiB_4_ structure, which exhibits two unit cells of type-**II** YPt_*x*_B_6–2*x*_, stacked along the *c* axis with further shifts of 12,12,0 with respect to each other. The boron atoms form columns of stacked truncated cubes with the yttrium atoms inside, by analogy to YB_6_. ELF distribution in this structure indicates covalent bonding within the [B_4_Pt_2_] polyanion, covalent B–B bonding between [B_4_Pt_2_] octahedra, and cationic character for yttrium. Existence of common structural fragments with hitherto reported boride structures strengthened the feasibility of the created structural models. Furthermore, the results of DFT studies of both type-**I** and type-**II** YPt_*x*_B_6–2*x*_ hint to the possibility of a formation of the material with a mixed structure due to the close values of minimized cell parameters. The value of electron density of states at the Fermi level of both structure variants is typical for metals (i.e., 1.4 and 1 eV^–1^/f.u. for type-**I** and type-**II** structures, respectively). Since the boron-rich compounds are hard refractory materials, the current work raises the question on further structural variants of CaB_6_ and AuCu_3_ intergrowth structures; the task employing TEM and quantum chemical computations is in progress.

## Supporting Information Available

Interatomic distances and coordination numbers in type-I and type-II YPt_*x*_B_6–2*x*_ structure models; coordination polyhedra of atoms in the YB_6_ and YPt_3_; coordination polyhedra of atoms in the type-I and type-II YPt_*x*_B_6–2*x*_ structure models and their short description; structural similarities among type-**I** YPt_*x*_B_6–2*x*_, LaB_2_C_2_, and CeB_2_C_2_ structures; structural similarities among type-**II** YPt_*x*_B_6–2*x*_, Zn_2_Ni_21_B_20_, and ErNiB_4_ structures; detailed visualization of electron localization function in the representative planes in the type-**I** and type-**II** YPt_*x*_B_6–2*x*_; densities of electron states of individual atoms in YB_6_ and YPt_3_; total energy of type-**I** and type-**II** YPt_*x*_B_6–2*x*_ as a function of cell parameters; partial electron densities of states of individual atoms in type-**I** and type-**II** YPt_*x*_B_6–2*x*_ calculated with and without SOC; results of phase analysis of alloys in the system Y–Pt–B in the vicinity of the YPt_*x*_B_6–2*x*_ phase at 780 °C obtained from Rietveld refinement of powder XRD; powder XRD patterns of most representative Y–Pt–B alloys annealed at 780 °C; and selected microstructures of Y–Pt–B alloys annealed at 780 °C (PDF)
