# Supplementary material for: Electronic and Structural Properties of MPtxB6–2x (M = Y, Yb): Structural Disorder in an Octahedral Boron Framework
Source: Inorg Chem. 2023 Nov 10;62(47):19164–77. doi: 10.1021/acs.inorgchem.3c01526 (PMC10685457; doi:10.1021/acs.inorgchem.3c01526)
Supplement: Supplementary file 1 — ic3c01526_si_001.pdf [file ic3c01526_si_001.pdf]

Supporting Information

Electronic and Structural Properties of MPt_xB_{6-2x} (M=Y, Yb). Structural Disorder in Octahedral Boron Framework

Leonid Salamakha[1,5,*], Oksana Sologub[1], Berthold Stöger[2], Gerald Giester[3], Peter F. Rogl[4], Herwig Michor[1], Ernst Bauer[1]

[1]Institute of Solid State Physics, TU Wien, A-1040 Vienna, Austria

[2]X-Ray Center, TU Wien, A-1060 Vienna, Austria

[3]Institute of Mineralogy and Crystallography, University of Vienna, A-1090 Vienna, Austria

[4]Institute of Materials Chemistry, University of Vienna, A-1090 Vienna, Austria

[5]Department of Physics of Metals, L'viv National University, L'viv, Ukraine

Table S1. Interatomic Distances (d, Å) and Coordination Numbers (CN) in Type-I and Type-II YPt_xB_{6-2x} structure models.

d (Å)	CN	*d* (Å)	CN	*d* (Å)	CN
type-**I** YPt_xB_{6-2x}					
Y1 - 4Pt1 2.8673<br>- 16B1 2.996	20	Pt1 - 8B1 2.335<br>Pt1 - 4Y1 2.8673	12	B1 - 2B1 1.64<br>B1 - 1B1 1.74<br>B1 - 2Pt1 2.335<br>B1 - 4Y1 2.996	9
type-**II** YPt_xB_{6-2x}					
Y1 - 8Pt1 2.8673<br>- 8B1 2.996	16	Y2 - 4Pt1 2.8673<br>- 8B1 2.996<br>- 8B2 2.996	20	Y3 - 8B1 2.996<br>Y3 - 16B2 2.996	24
Pt1 - 4B1 2.335<br>Pt1 - 2B2 2.335<br>Pt1 - 2Pt1 2.8673<br>Pt1 - 2Y1 2.8673<br>Pt1 - 2Y2 2.8673	12	B1 - 2B2 1.64<br>B1 - 1B1 1.74<br>B1 - 2Pt1 2.335<br>B1 - 1Y1 2.996<br>B1 - 2Y2 2.996<br>B1 - 1Y3 2.996	9	B2 - 2B1 1.64<br>B2 - 1B2 1.64<br>B2 - 1B2 1.74<br>B2 - 1Pt1 2.335<br>B2 - 2Y2 2.996<br>B2 - 2Y3 2.996	9

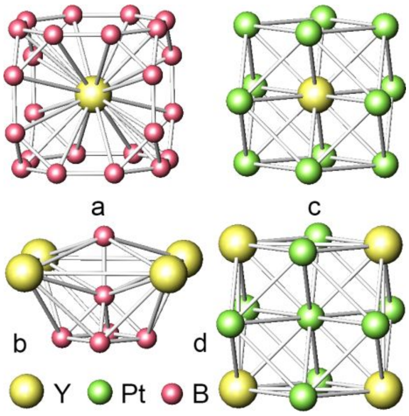

Figure S1. Coordination polyhedra of atoms in the YB_6 (a - [YB_{24}], b - [BB_5Y_4]) and YPt_3 (c - [YPt_{12}], d - [$PtPt_8Y_4$]).

Coordination environment of Y and Pt atoms in type-I and type-II models of YPt_xB_{6-2x} structure.

Y in the type-**I** structure of YPt_xB_{6-2x} is coordinated by 20 atoms; its coordination polyhedron derives from the $[YB_{24}]$ polyhedron in YB_6, but includes four platinum atoms instead of eight boron atoms (Figure S2a, S1a). Platinum is situated inside a cubooctahedron $[PtB_8Y_4]$ (Figure S2b). Upon transformation to the type-**II** YPt_xB_{6-2x} structure model, the Y site of the disordered structure splits into three independent crystallographic atom positions (Section 3.3, Table 5) among which i) the site Y3 is coordinated by 24 boron atoms arranged in a way to form a truncated cube (thus corresponding to the coordination polyhedron of Y in YB_6) (Figure S3c, S1a); ii) the coordination environment of Y2 is similar to the coordination polyhedron of Y in type-**I** YPt_xB_{6-2x}, but includes two different sorts of boron atoms (Figure S3b); iii) the coordination polyhedron around Y1 exhibits 16 vertices and is reminiscent to the cubooctahedron of Y in YPt_3 in which, however, four platinum atoms are replaced by eight boron atoms (Figure S3a, Figure S1a). Platinum atoms in type-**II** YPt_xB_{6-2x} are found at the centres of distorted cubooctahedra, the vertices of which are formed by four Y, two Pt and six B atoms (Figure S3d).

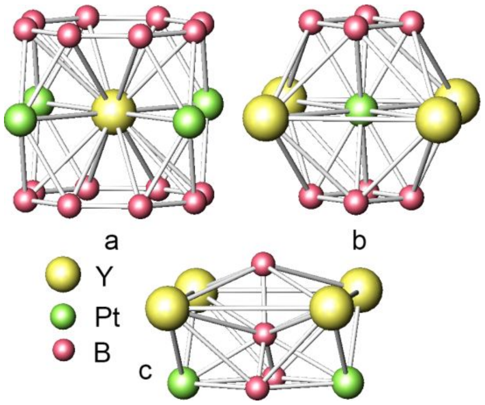

Figure S2. Coordination polyhedra of atoms in the type-**I** YPt_xB_{6-2x} structure model (a - $[YB_{16}Pt_4]$, b - $[PtB_8Y_4]$, c - $[BB_3Pt_2Y_4]$).

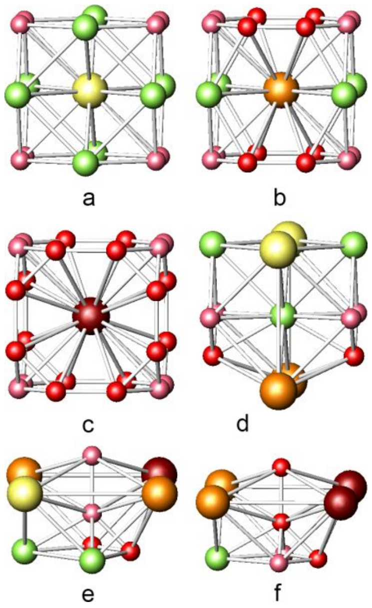

Figure S3. Coordination polyhedra of atoms in the type-**II** YPt_xB_{6-2x} structure model: a - [$Y1B_8Pt_8$], b - [$Y2B_{16}Pt_4$], c - [$Y3B_{24}$], d - [$Pt1B_6Pt_2Y_4$], e - [$B1B_3Pt_2Y_4$], f - [$B2B_4Pt_1Y_4$]. Gold balls represent Y1 atoms, orange balls indicate Y2 atoms, brown balls stay for Y3 atoms, green ball represent Pt1 atoms, light red balls and red balls represent B1 and B2 atoms respectively.

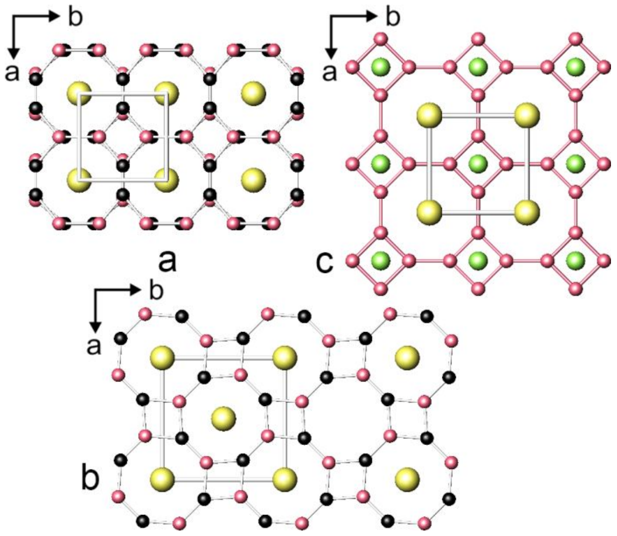

Figure S4. 4.8^2 B/C nets in the crystal structures of LaB_2C_2 (space group *P*4_2/*mmc*) (a) and CeB_2C_2 (space group *P*4/*mbm*) (b) in comparison with the 4.8^2 boron nets in the type-**I** YPt_xB_{6-2x} structure model (c). Gold balls correspond to Y, RE atoms; green balls stay for Pt; red and black balls present boron and carbon atoms.

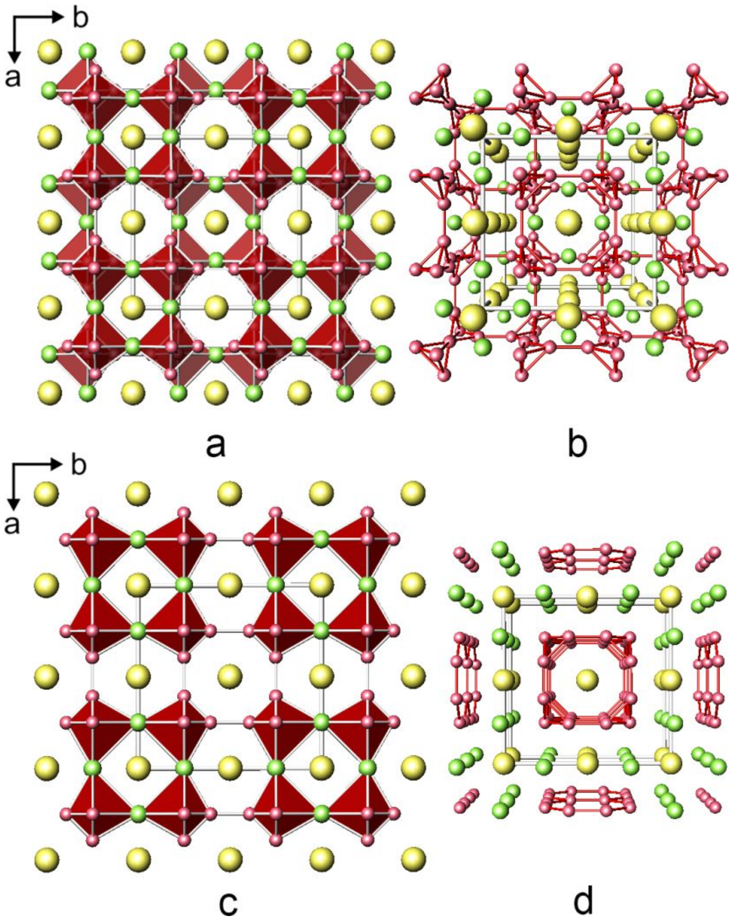

Figure S5. Arrangement of [B_4Ni_2] and [B_4Pt_2] octahedra in the structures $ErNiB_4$-type (space group *I*4/*mmm*) (a) and type-**II** YPt_xB_{6-2x} (*Pm*-3*m*) (c). Network of boron atoms in $ErNiB_4$ (b) and the columns of boron truncated cubes in type-**II** YPt_xB_{6-2x} (d). Gold balls correspond to Y, RE atoms; green balls stay for Ni, Pt; red balls present boron atoms.

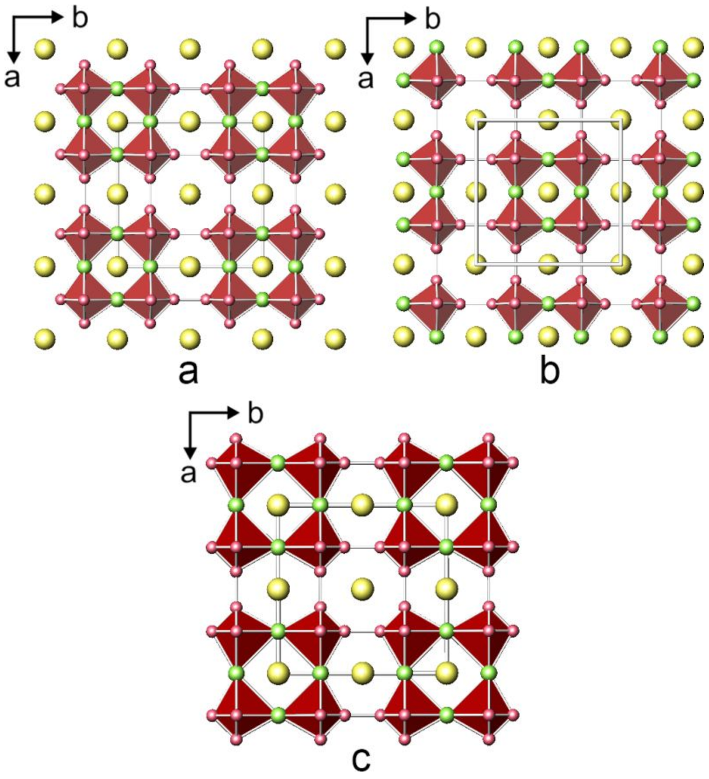

Figure S6. Two structural blocks, stacked along *c* in the $ErNiB_4$-type structure (a, b) in comparison with the structure model of type-**II** YPt_xB_{6-2x} (c). Gold balls correspond to Y, RE atoms, green balls stay for Ni, Pt, and red balls present boron atoms.

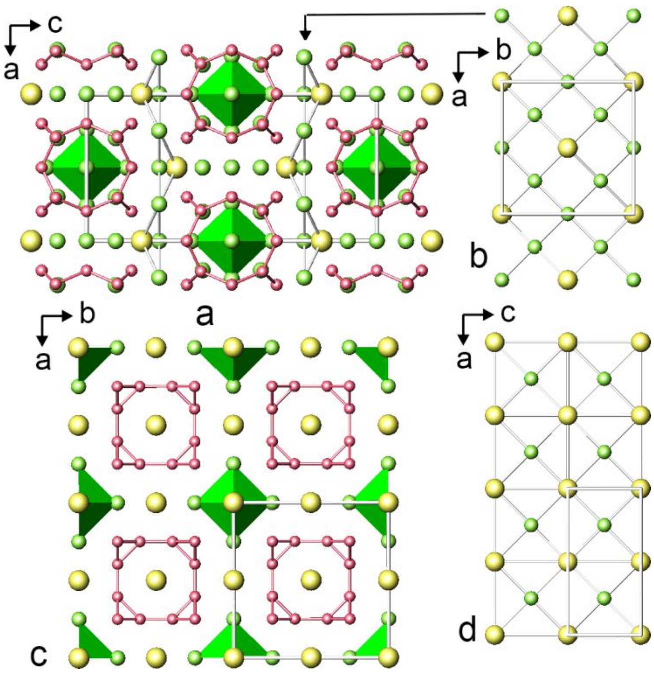

Figure S7. Crystal structure of $Zn_2Ni_{21}B_{20}$ (a) and type-**II** YPt_xB_{6-2x} (c). Planar layer formed by intercrossed chains of Ni (b). Planar metalic layer in the type-**II** YPt_xB_{6-2x} (d).

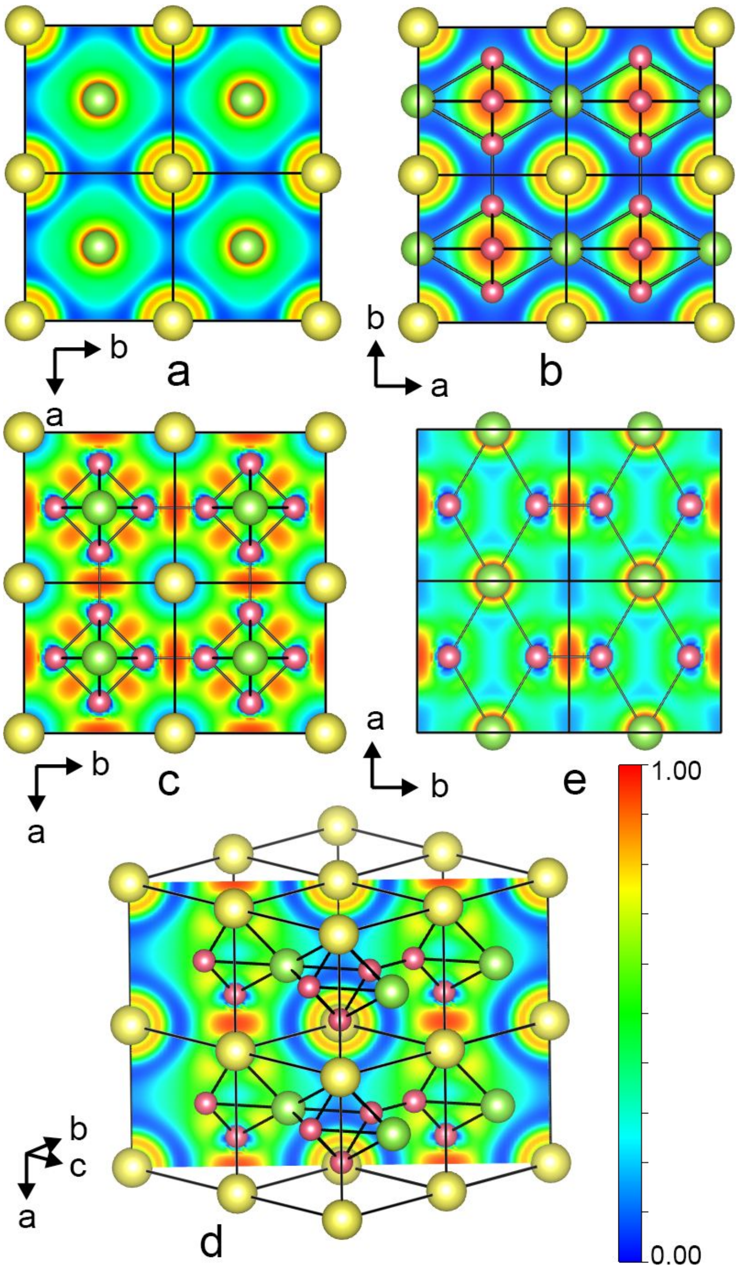

Figure S8. (a) Sections of calculated electron localization function distribution in the type-**I** YPt_xB_{6-2x} (x=1.15) within the planes: (001) showing Y1-Pt1 interaction; (b) (0-10) bearing Y1 atoms (Pt and B atoms are out of plane); (c) (002) revealing B1-B1 interactions (Y and Pt atoms are out of plane); (d) (0(-1)1) visualizing Y1-B1 interaction (Pt and some B atoms are out of plane); (e) (200) showing the interaction of Pt1-B1 and B1-B1 in [B_4] squares. Four unit cells are shown. Y1 - gold balls, Pt1 - green ball, B1 - light red balls.

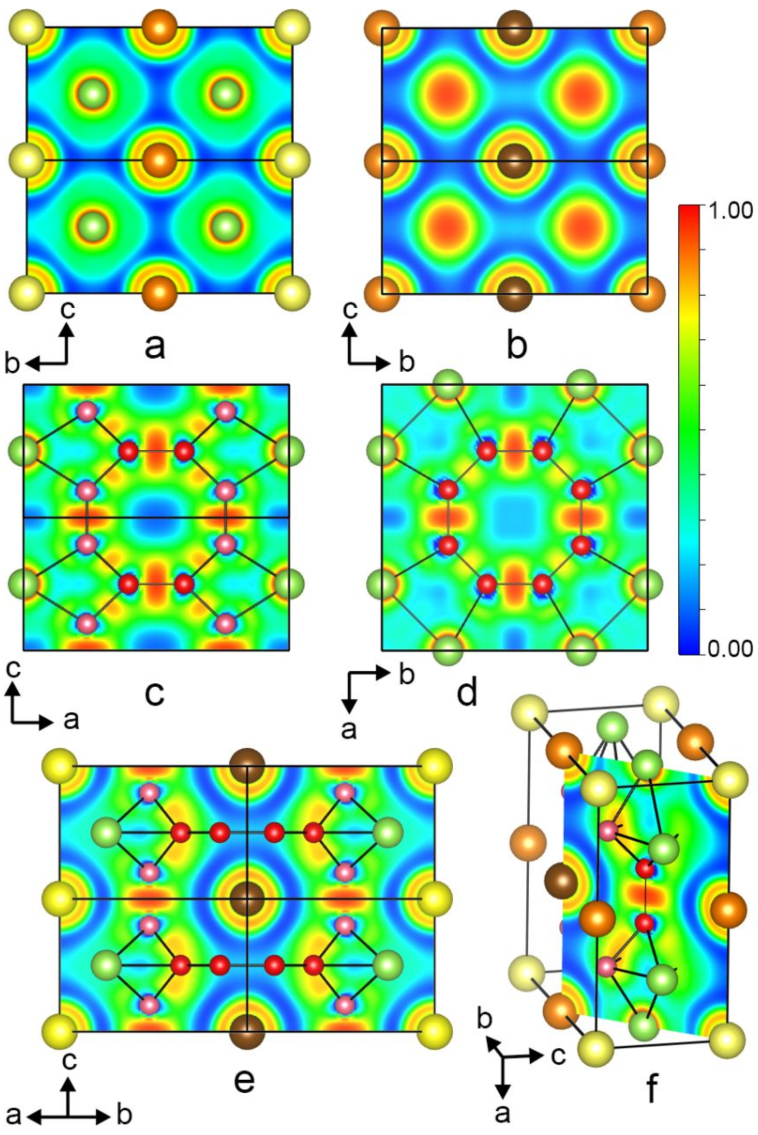

Figure S9. Sections of calculated electron localization function in the type-**II** YPt_xB_{6-2x} (x=1.15) in the planes: (a) (100) showing Y1-Pt1 and Y2-Pt2 interactions; (b) (200) bearing Y2 and Y3 atoms; (c) (040) revealing Pt1-B1, B1-$B1_{out}$ (interoctahedral) and B2-$B2_{out}$ (interoctahedral) interactions; (d) (002) visualizing Pt1-Pt1, Pt1-B2 and B2-$B2_{out}$ (interoctahedral); (e) (110) presenting the Y1-B1, Y3-B1 and B1-B1 interactions. The maxima of ELF are also shown for B2-B1-B2 triangles; (f) (021) showing ELF maxima for bonds between Y2-B2, Y3-B2, B2-$B2_{out}$ and within B2-B1-B2 triangles. Two unit cells are shown in (a), (b), (c), (d) and (e). Y1 - gold balls, Y2 - orange balls, Y3 - brown balls, Pt1 - green ball, B1 - light red balls, B2 - red balls.

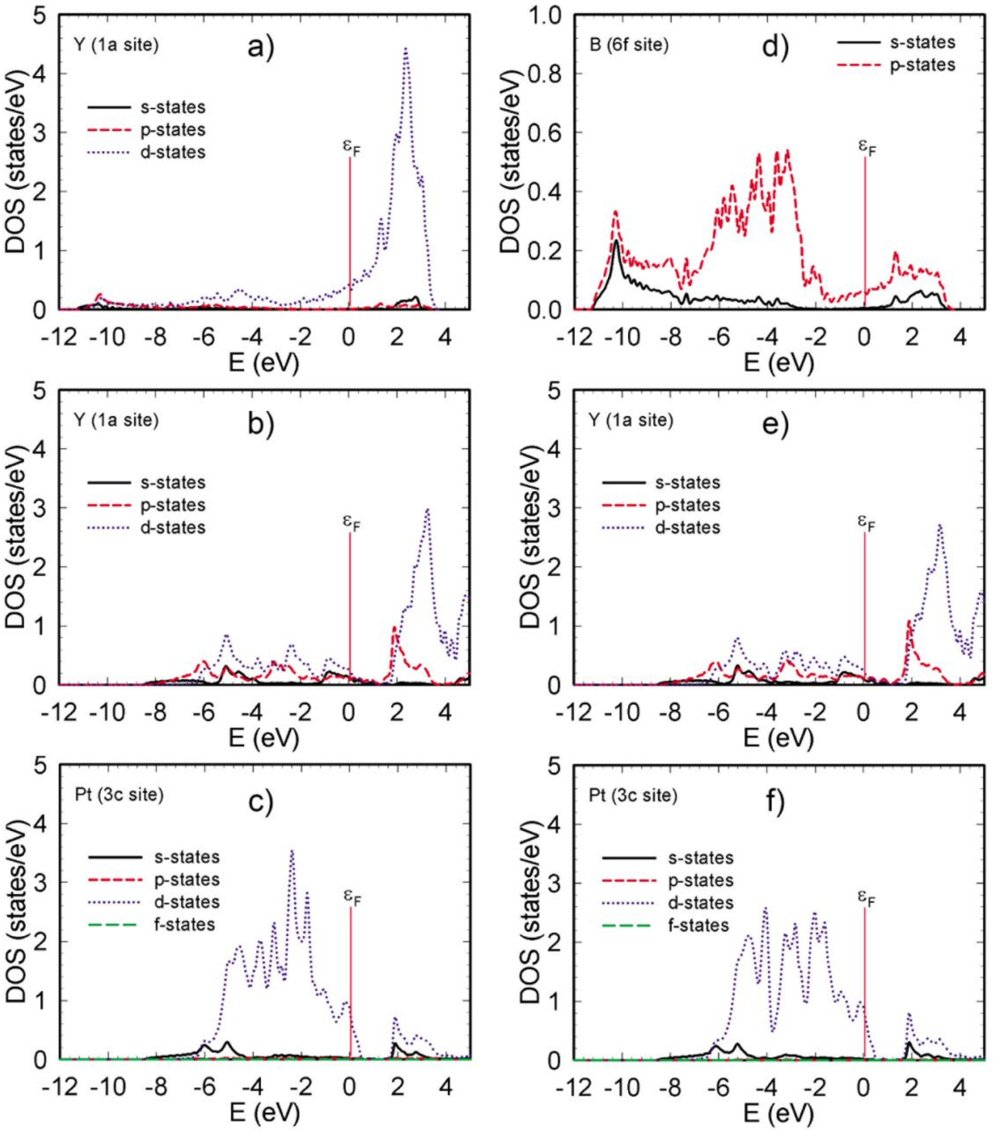

Figure S10. Densities of electron states of individual atoms in YB_6 calculated without spin-orbit coupling (a, d) and YPt_3 in both non-SOC (b, e) and SOC (c, f) cases.

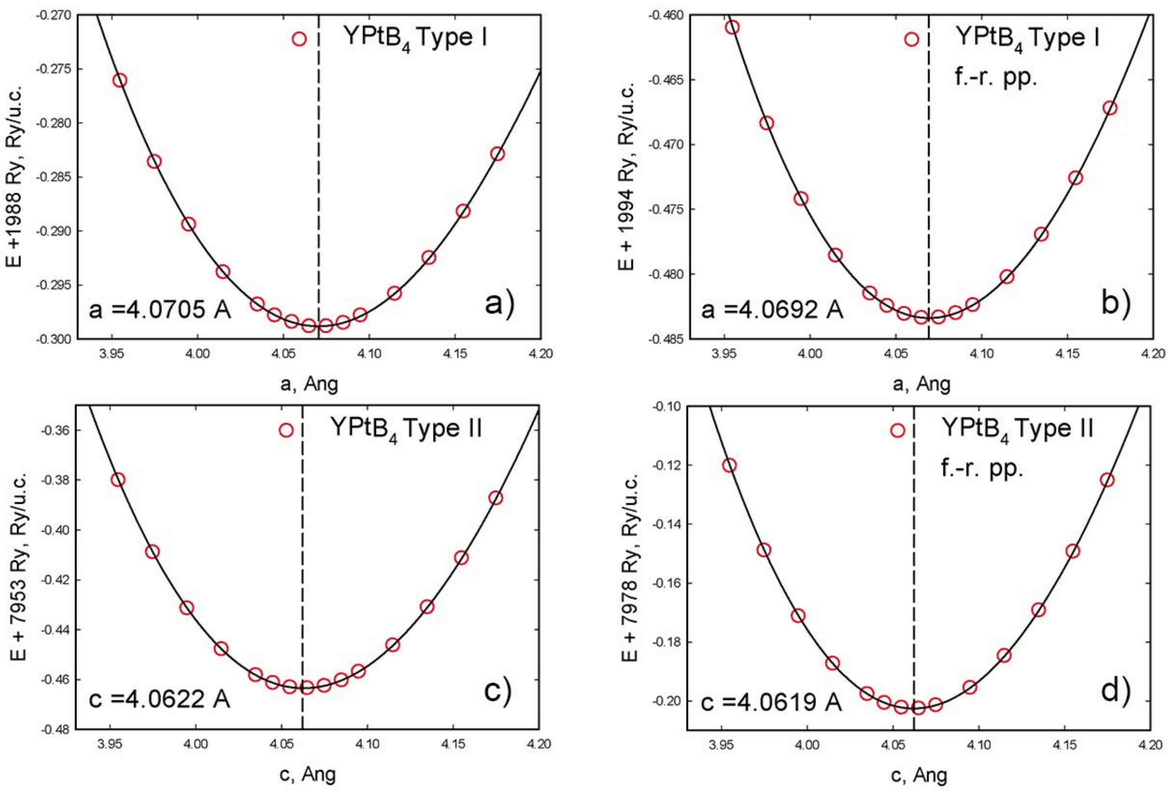

Figure S11. Total energy of type-**I** and type-**II** YPt_xB_{6-2x} calculated without (a, c) and with (b, d) SOC as a function of cell parameters. Solid lines in figures are guide for the eye.

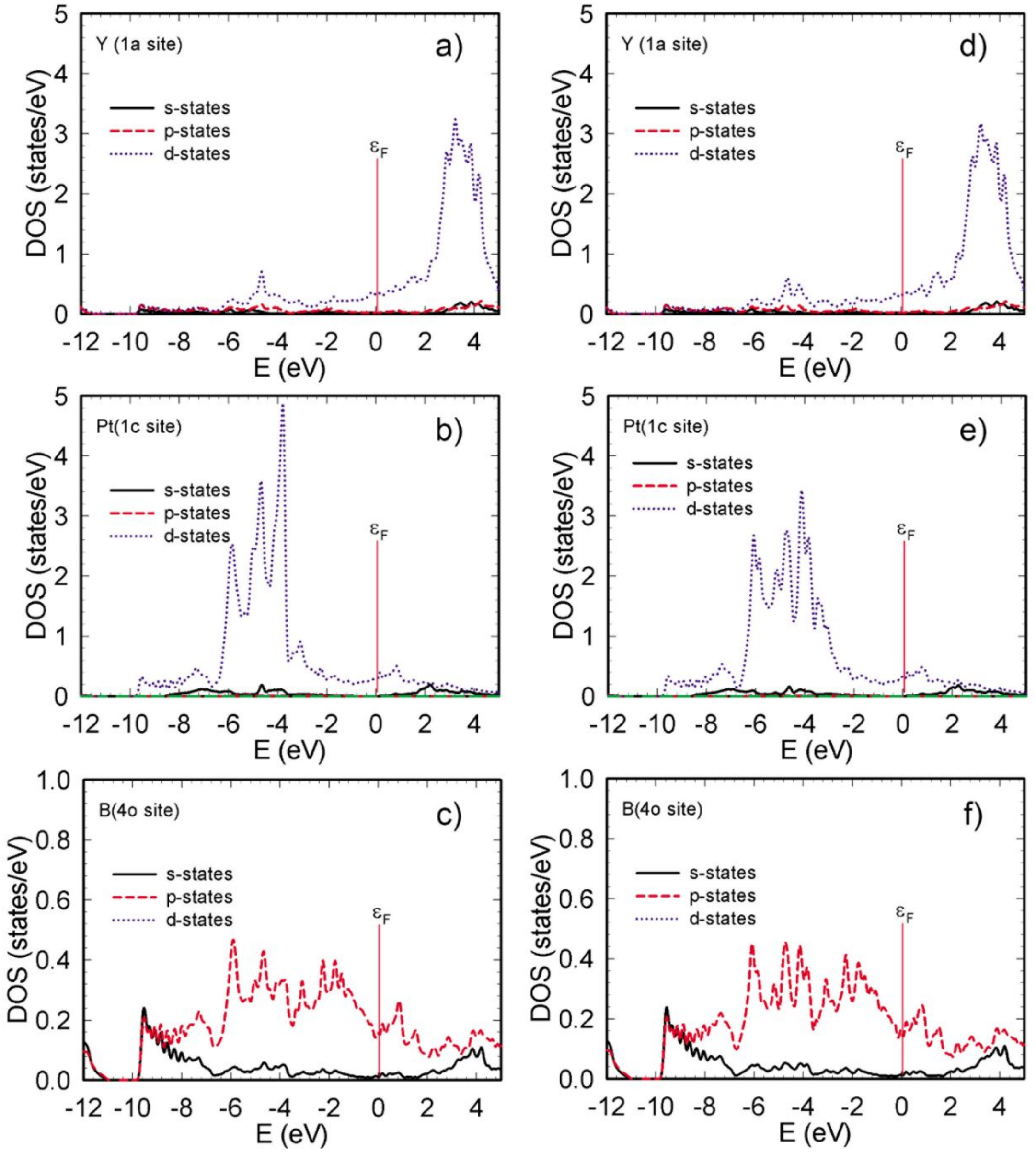

Figure S12. Partial electron densities of states of individual atoms in type-**I** YPt_xB_{6-2x} calculated without SOC (a, b, c) and with SOC (d, e, f).

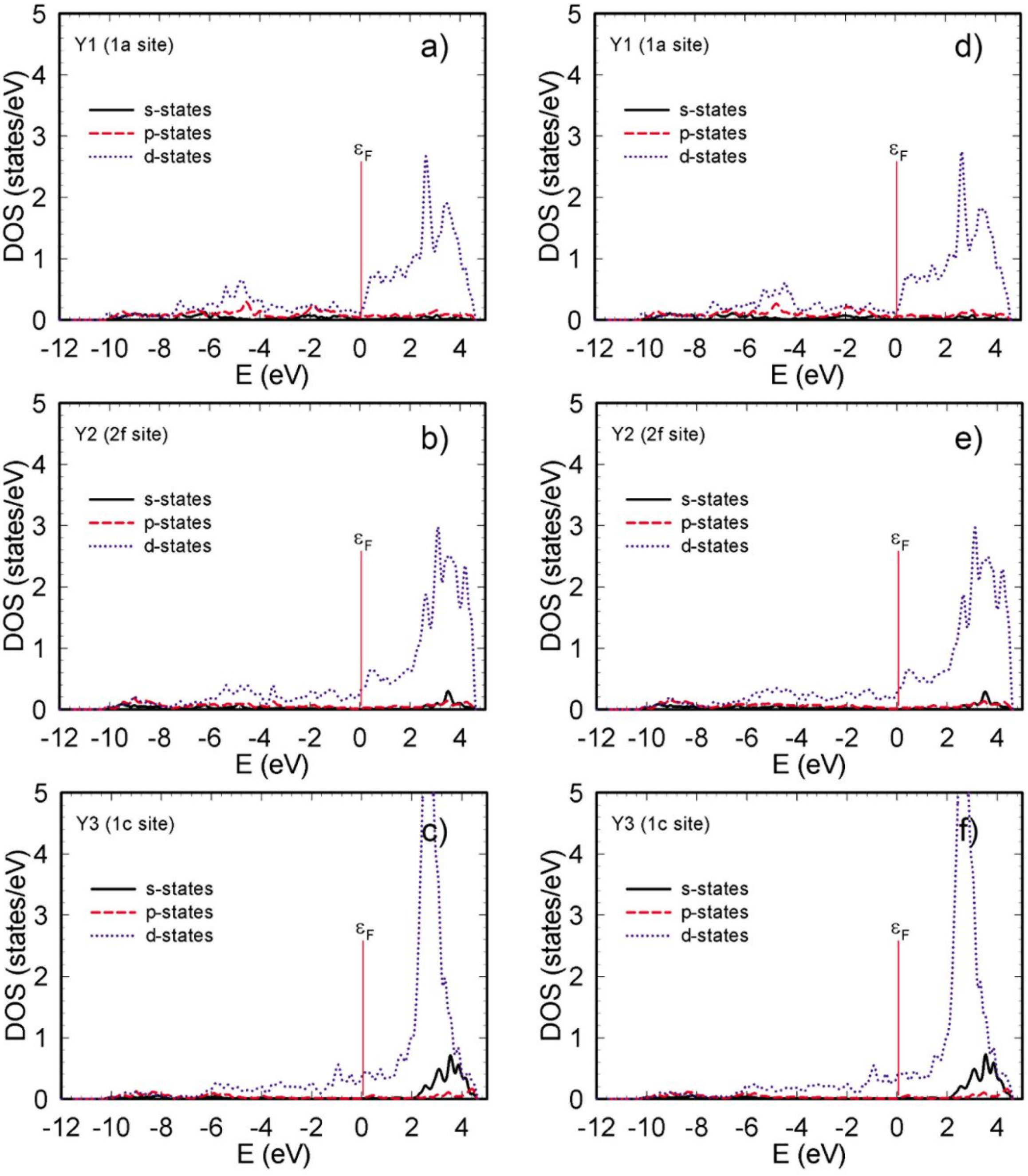

Figure S13. Partial electron densities of states of Y atoms in type-**II** YPt_xB_{6-2x} calculated without SOC (a, b, c) and with SOC (d, e, f).

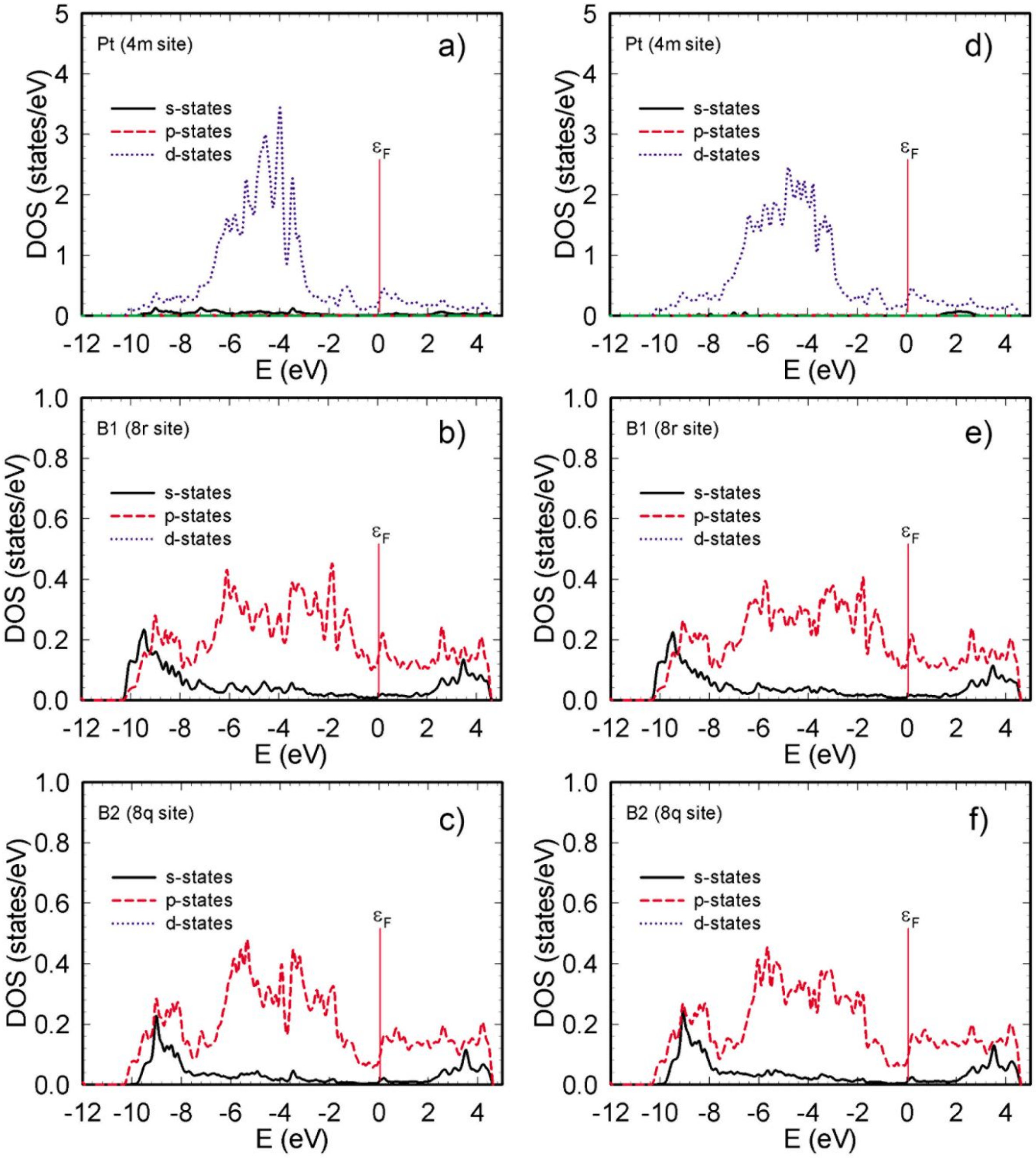

Figure S14. Partial electron densities of states of individual Pt and B atoms in type-**II** YPt$_x$B$_{6-2x}$ calculated without SOC (a, b, c) and with SOC (d, e, f).

Table S2. Phase Analysis of Alloys in the System Y-Pt-B in the Vicinity of YPt_xB_{6-2x} Phase at 780 °C.

Sample number[a]	Nominal sample composition (at. %)			X-ray phase analysis	Lattice parameters, Å		
	Y	Pt	B		a	b	c
1	10		90	YB_{12}<br>YB_6	7.50350(8)<br>4.10332(5)		
2	16		84	YB_6<br>YB_4<br>YB_{12} (traces)	4.10030(4)<br>7.1009(1)<br>7.4988(1)		 4.0102(1)
3	17	4	79	YB_6<br>YB_4<br>YPt_xB_{6-2x}	4.09615(5)<br>7.1037(1)<br>4.06772(6)		 4.0176(1)
4	14	6	80	YPt_xB_{6-2x}<br>YB_6<br>unknown	4.0550(1)<br>4.09656(9)<br>traces		
5	15	19	66	YPt_xB_{6-2x}<br>YPt_5B_2<br><br>unknown	4.05599(8)<br>15.4873(5)<br><br>traces	 5.5453(2)<br>105.047(3)	 5.5684(2)
6	15	25	60	YPt_xB_{6-2x}<br>YPt_5B_2	4.05626(7)<br>15.4927(4)	 5.5480(1)<br>105.028(1)	 5.5693(1)
7	14	54	32	YPt_5B_2<br><br>YPt_xB_{6-2x}	15.4936(2)<br><br>4.05159(5)	5.54526(7)<br>105.099(1)	5.56957(7)
8	16	54	30	YPt_3B<br>YPt_xB_{6-2x}<br>YPt_5B_2	3.9484(1)<br>4.05023(9)<br>15.4870(4)	 <br>5.5462(1)<br>105.089(1)	4.9909(2)<br><br>5.5686(2)
9	18.5	37	44.5	YPt_xB_{6-2x}<br>YPt_2B<br>YPt_3B	4.05093(6)<br>5.2996(1)<br>3.94496(9)		 7.8829(2)<br>4.9860(2)
10	20	8	72	YB_4<br>YPt_xB_{6-2x}<br>YPt_2B	7.10435(7)<br>4.05081(6)<br>5.30531(9)		4.01878(5)<br><br>7.8819(2)
11	21	13	66	YB_4<br>YPt_2B<br>YPt_xB_{6-2x}<br>(traces)	7.10379(6)<br>5.30245(5)<br>4.0514(7)		4.01884(4)<br>7.8894(1)
12	17	19.5	63.5	YPt_xB_{6-2x}	4.05309(3)		

[a] corresponds to the numbers in Figure 9.

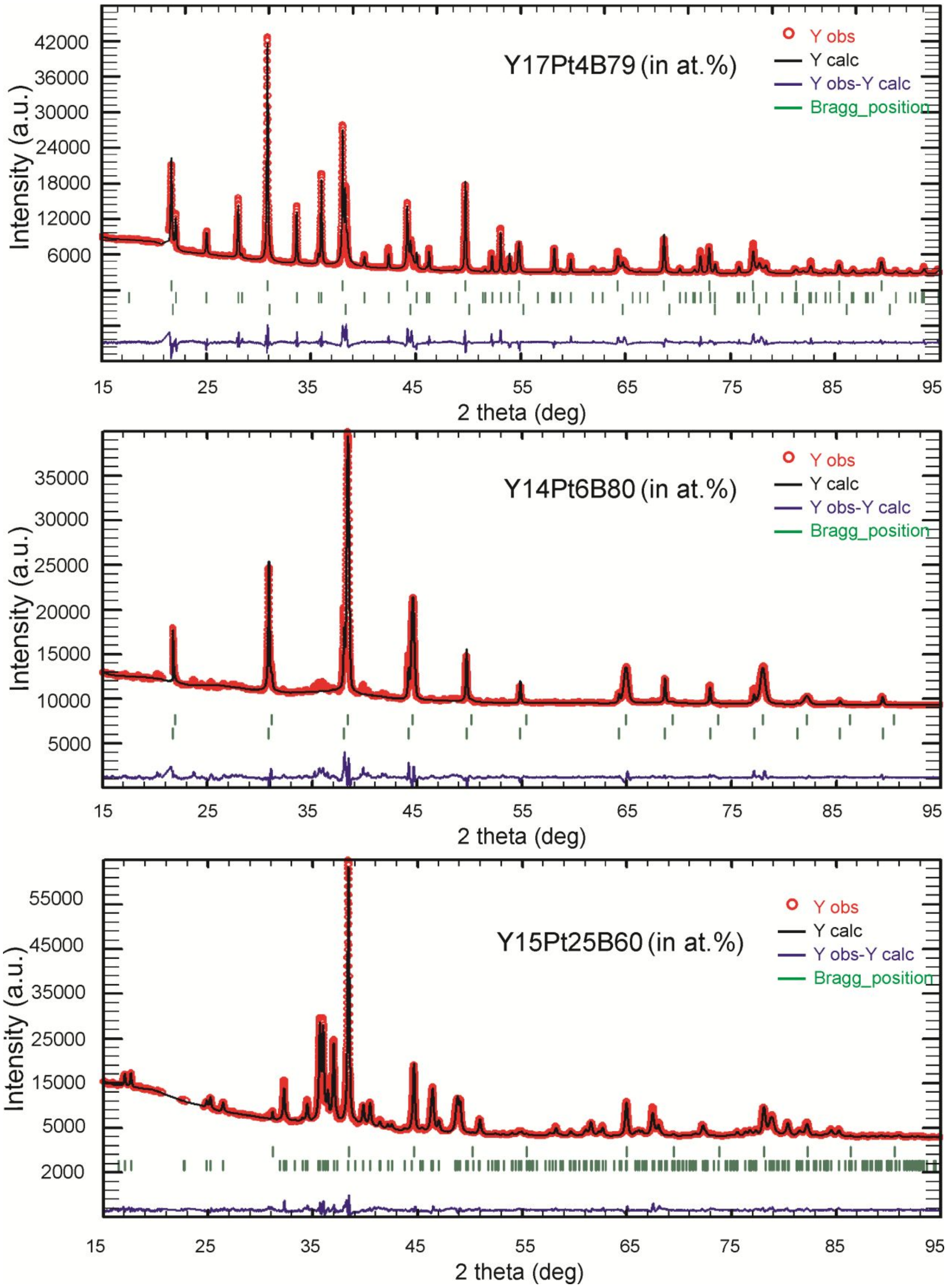

Figure S15. Powder X-ray diffraction patterns of Y-Pt-B alloys annealed at 780 ºC: no.3 $Y_{17}Pt_4B_{79}$ (in at.%) (upper row of *hkl* labels corresponds to YB_6, middle row stands for YPt_xB_{6-2x}, lower row represents the phase YB_4), no.4 $Y_{14}Pt_6B_{80}$ (in at.%) (upper row of *hkl* labels stands for the phase YPt_xB_{6-2x}, lower row represents YB_6, un-indexed reflexes correspond to the traces of unknown phase); no.6 $Y_{15}Pt_{25}B_{60}$ (in at.%) (upper row of *hkl* labels stands for the phase YPt_xB_{6-2x}, lower row represents the YPt_5B_2).

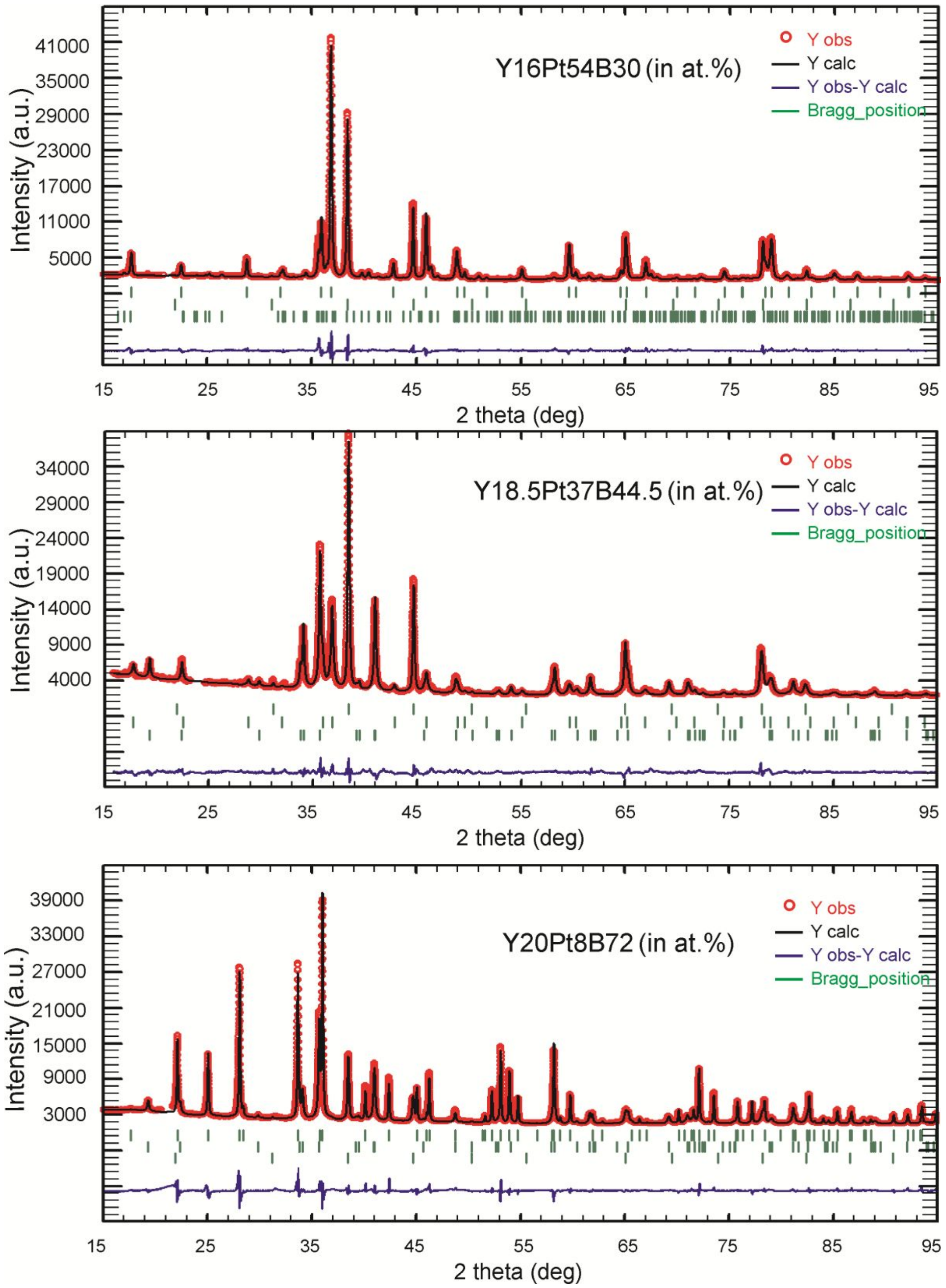

Figure S16. Powder X-ray diffraction patterns of Y-Pt-B alloys annealed at 780 ºC: no.8 $Y_{16}Pt_{54}B_{30}$ (in at.%) (upper row of *hkl* labels corresponds to YPt_3B, middle row stands for YPt_xB_{6-2x}, lower row represents the phase YPt_5B_2), no.9 $Y_{18.5}Pt_{37}B_{44.5}$ (in at.%) (upper row of *hkl* labels corresponds to YPt_xB_{6-2x}, middle row stands for YPt_3B, lower row represents the phase YPt_2B); no.10 $Y_{20}Pt_8B_{72}$ (in at.%) (upper row of *hkl* labels corresponds to YB_4, middle row stands for YPt_2B, lower row represents the phase YPt_xB_{6-2x}).

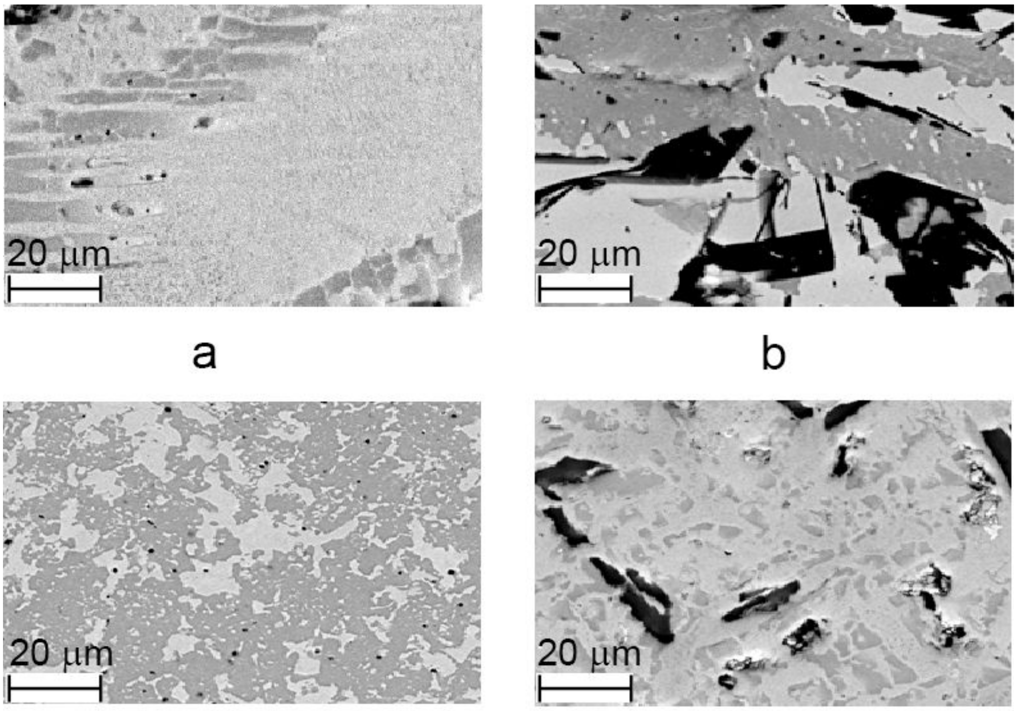

a b c d

Figure S17. Selected microstructures of Y-Pt-B alloys annealed at 780 ºC: (a) $Y_{16.7}Pt_{16.6}B_{66.7}$ showing YPt_xB_{6-2x} phase; contrast is attributed to grain orientation; (b) $Y_{15}Pt_{19}B_{66}$ (light grey - YPt_5B_2, dark grey - YPt_xB_{6-2x}, black - unknown boron rich phase; (c) $Y_{18}Pt_{42}B_{40}$ (light grey - YPt_3B, medium grey - YPt_xB_{6-2x}); (d) $Y_{22}Pt_{36}B_{42}$ (light grey - YPt_2B, medium grey - YPt_xB_{6-2x}, black - YB_4(Pt)).
